# Do health policies address the availability, accessibility, acceptability, and quality of human resources for health? Analysis over three decades of National Health Policy of India

**DOI:** 10.1186/s12960-021-00681-1

**Published:** 2021-11-13

**Authors:** Sweta Dubey, Jeel Vasa, Siddhesh Zadey

**Affiliations:** 1 Association for Socially Applicable Research (ASAR), Pune, Maharashtra India; 2 Government Medical College and Hospital, Nagpur, Maharashtra India; 3 Rajarshi Chhatrapati Shahu Maharaj Government Medical College, Kolhapur, Maharashtra India; 4 Duke Global Health Institute, Duke University, Durham, NC USA; 5 Department of Surgery, Duke University School of Medicine, Durham, NC USA

**Keywords:** Human Resources for Health, India, Policy analysis, Health system strengthening, Deficit indices, National Health Policy

## Abstract

**Background:**

Human Resources for Health (HRH) are crucial for improving health services coverage and population health outcomes. The World Health Organisation (WHO) promotes countries to formulate holistic policies that focus on four HRH dimensions—availability, accessibility, acceptability, and quality (AAAQ). The status of these dimensions and their incorporation in the National Health Policies of India (NHPIs) are not well known.

**Methods:**

We created a multilevel framework of strategies and actions directed to improve AAAQ HRH dimensions. HRH-related recommendations of NHPI—1983, 2002, and 2017 were classified according to targeted dimensions and cadres using the framework. We identified the dimensions and cadres focussed by NHPIs using the number of mentions. Furthermore, we introduce a family of dimensionwise deficit indices formulated to assess situational HRH deficiencies for census years (1981, 2001, and 2011) and over-year trends. Finally, we evaluated whether or not the HRH recommendations in NHPIs addressed the deficient cadres and dimensions of the pre-NHPI census years.

**Results:**

NHPIs focused more on HRH availability and quality compared to accessibility and acceptability. Doctors were prioritized over auxiliary nurses-midwives and pharmacists in terms of total recommendations. AAAQ indices showed deficits in all dimensions for almost all HRH cadres over the years. All deficit indices show a general decreasing trend from 1981 to 2011 except for the accessibility deficit. The recommendations in NHPIs did not correspond to the situational deficits in many instances indicating a policy priority mismatch.

**Conclusion:**

India needs to incorporate AAAQ dimensions in its policies and monitor their progress. The framework and indices-based approach can help identify the gaps between targeted and needed dimensions and cadres for effective HRH strengthening. At the global level, the application of framework and indices will allow a comparison of the strengths and weaknesses of HRH-related policies of various nations.

**Supplementary Information:**

The online version contains supplementary material available at 10.1186/s12960-021-00681-1.

## Background

Human Resources for Health (HRH) are “all people primarily engaged in actions with the primary intent of enhancing health” [1]. HRH is a crucial component of health systems to improve health services coverage and population health outcomes [2]. HRH strengthening is, therefore, quintessential to achieve Universal Health Coverage (UHC) [3] within the broader Sustainable Development Goals (SDGs) framework [4]. However, there is a shortage of 6.9 million and 4.2 million skilled HRH in South–East Asia and Africa, respectively [5]. To address this shortage, under SDG-3.c, the World Health Organization (WHO) encourages nations with an HRH crisis to create and implement national and local policies focused on four HRH dimensions—availability, accessibility, acceptability, and quality (AAAQ) [5]. Availability refers to adequate HRH supply and stock corresponding to population health needs. Accessibility is the equitable spatial, temporal, organizational, and financial access to HRH. Acceptability is determined by HRH characteristics, such as sex and age composition, skills mix, and cultural awareness that can match the population expectations. Finally, quality refers to the competencies, skills, knowledge, and professional behavior of health workers.

India is facing a critical shortage of HRH with only 160 skilled health workers per 100,000 people [6]. In 2016, 36% of total HRH served in rural areas which had a 71% population with doctors and nurses constituting the largest portion of HRH [7]. In response to global calls and the existing crisis, India adopted the target of achieving 450 physicians, nurses, and midwives per 100,000 population by 2030 under SDG indicator 3.c [8]. The National Health Policy of India (NHPI), arguably, the most comprehensive policy securing the health of a billion Indians, encompasses recommendations and plans to attain UHC [9]. The first NHPI (1983) was framed as a response to the 1978 Alma Ata Declaration. Subsequently, NHPI was revised in 2002 and 2017 to match the country’s needs and progress. Integrating HRH recommendations aimed at AAAQ dimensions in NHPI can bridge national priority-setting and global calls for action.

Analysis of NHPIs in the context of HRH is crucial for the following reasons. First, it helps to study policy changes and their effects on HRH strengthening over an extensive period of three decades. Second, it helps to evaluate the nation’s policy commitment towards addressing HRH needs and achieving development targets. Third, it could guide the development of future policies geared towards areas of need in an evidence-based fashion. Thus, our analysis has three main aims:

1. Identify the degree of incorporation of WHO’s AAAQ dimensions in NHPI’s HRH-related recommendations.
2. Identify AAAQ dimensional deficiencies in HRH cadres before three NHPI adoptions to gauge situational needs and also assess longitudinal trends in deficiencies.
3. Investigate whether NHPI recommendations targeted the deficient HRH dimensions and cadres.

## Methods

### Framework for policy content analysis

**Fig. 1:**
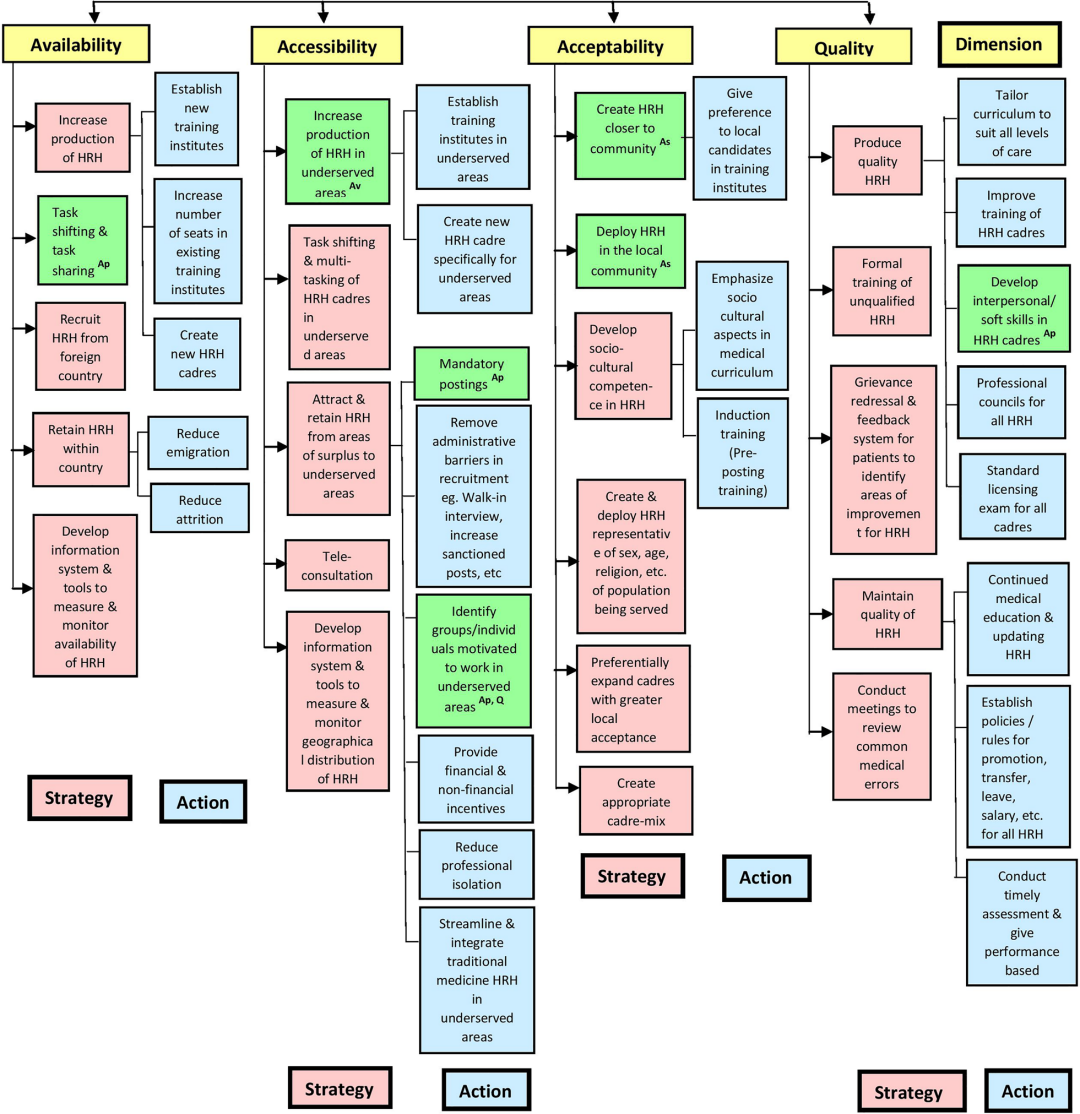
Framework of strategies and actions for strengthening HRH availability, accessibility, acceptability, and quality dimensions. *HRH* Human Resources for Health. Strategies/actions highlighted in green apply to more than one dimension. Shared dimension(s) are mentioned at the end of the highlighted strategies/actions as abbreviation(s) in the superscript. *Av* availability, *As* accessibility, *Ap* acceptability, *Q* quality

We created a three-tiered framework with dimensions, strategies, and actions for assessing HRH strengthening (Fig. 1). The first level is based on four HRH–AAAQ dimensions proposed by the WHO—Global Health Workforce Alliance (GHWA) [10]. GHWA is a partnership of global stakeholders administered by WHO dedicated to mitigating the HRH crisis. The ‘No Health Without a Workforce’ report [10] defines availability as the sufficient supply and stock of HRH corresponding to the health needs of the population; accessibility as the equitable spatial, temporal, organizational, and financial access to HRH; acceptability as the characteristics of HRH to meet the expectations of patients in terms of its profile, sex and age composition, its skills mix, and cultural awareness; and quality as the competencies, skills, knowledge, and behavior of the health worker as assessed according to professional norms and as perceived by users [11].

We conceptualized the second (strategies) and third (actions) levels of the framework under the dimensions. We define strategies as the broad approaches that can be used independently or in combination to improve a dimension. We define actions under strategies as directly implementable measures to produce the desired improvement. For example, to improve the HRH availability, one strategy is increasing production that can be achieved by applying either one or all of the three actions—increasing the number of training institutes, increasing the intake capacity of existing institutes, and creating new cadres (e.g., rural-specific doctors). Certain strategies and actions can improve more than one dimension, e.g., creation and deployment of HRH closer to community can enhance acceptability as well as accessibility, introduction of new cadres for underserved areas can enhance both accessibility and acceptability. Such multidimensional strategies and actions are highlighted in Fig. 1. Based on an iterative scoping review of the literature [12–14] (Additional file 1. a), relevant strategies and actions that could improve corresponding dimensions were added to the framework. The proposed framework has 4 dimensions, 21 strategies, and 24 actions (Fig. 1).

### Data sources and variables

We used the National Health Portal of India [15] to obtain policies and committee reports. The National Health Portal is set up by the Ministry of Health and Family Welfare (MoHFW), India to serve as a single point of access for consolidated healthcare-related information for citizens. We obtained NHPI—1983, 2002, and 2017 reports from the Portal’s ‘Policy’ section to extract cadrewise HRH-related recommendations. We obtained the Health Survey & Development (Bhore) Committee (1946) [16] and High-level Expert Group (HLEG) (2011) [17] reports from the ‘Committees and Commissions’ section of the Portal. We calculated two cadrewise requirement thresholds, HRH per 100,000 population—R-1 and R-2 using Bhore and HLEG reports, respectively. The requirement threshold values are given in Table 1 and the calculation details are presented in the Additional file 1. b. We chose the Bhore Committee and HLEG reports as the former present the first HRH requirement thresholds for Independent India, while the latter presents the most recent. The Bhore Committee set an ambitious target for the then India, which would have brought India closer to UHC much earlier. The HLEG presents a recently relevant and realistic target-setting, considering the existing HRH shortage and global development calls.

**Table 1:** Cadrewise requirement thresholds from Bhore Committee and High-level Expert Group (HLEG) per 100,000

Cadres	Requirement Thresholds as per Bhore Committee Report	Requirement Thresholds as per HLEG Committee Report
ANM	60	73.72
Nurse	355.91	112.26
Pharmacist	43.97	22.5
AYUSH	NA	6.45
Dentist	20	6.73
Doctor	121.78	33.5

*ANM* auxiliary nurse-midwife, *AYUSH*: Ayurveda, Yoga and Naturopathy, Unani, Siddha and Homeopathy, *NA* not available

Data on HRH cadre numbers were taken from the Census of India and National Sample Survey (NSS). We included allopathic doctors (referred to as doctors in the rest of the paper), Ayurveda, Yoga and Naturopathy, Unani, Siddha and Homeopathy (AYUSH) practitioners, nurses, auxiliary nurse-midwives (ANMs), dentists, and pharmacists. The National Occupational Classification (NOC) [18] codes were used to classify self-reported occupations from the Census and NSS (Additional file 2: Table S1). NOC-1968 was used for analyzing 1981 data and NOC-2004 for 2001 and 2011 data. We extracted the absolute number of male and female personnel of each cadre for rural, urban, and total groups at the national level from the Census of India [19] for census years immediately before each NHPI release ie. 1981 [20], 2001 [21], and 2011 [22] (Additional file 2: Table S2). The absolute numbers were used to get cadrewise present HRH densities (P) per 100,000 for 1981, 2001, 2011 using the respective census populations (Additional file 1.b). NSS ‘Employment and Unemployment’ rounds [23] were used to extract the qualified HRH data. These surveys are nationally representative multistage, stratified, cluster sample surveys that collect self-reported information on employment, level of education, among other attributes. Similar to previous studies [7, 24], we compared the educational achievements of self-reported health workers in the 38th (January to December 1983) [25], 61st (July 2004–June 2005) [26], and 68th (July 2011–June 2012) [27] NSS rounds with the required qualifications recommended by registration institutes and councils for each cadre, e.g., Medical Council of India for allopathic doctors, Indian Nursing Council for nurses and ANMs, etc. (Additional file 2: Table S3). The proportions of qualified HRH obtained from NSS 38th, 61st, and 68th rounds of NSS were applied to the 1981, 2001, and 2011 census absolute HRH values, respectively, to get the qualified HRH relative to the overall HRH as a proxy of HRH quality (see ahead). Although the census years 1981 and 2001 do not perfectly match the 38th (1983) and 61st (2004–05) NSS rounds by the calendar year, the calculation of qualified HRH using the above method is valid assuming that the proportions would not change in small periods of 2–4 years.

### Indices for dimensional HRH deficits

The WHO–GHWA report [10] has suggested a few matrices for measuring AAAQ—the density of skilled health professionals per 10,000 population for availability; the geographic (rural–urban) distribution of physicians for accessibility; the nurses to doctors ratio as well as the proportion of female physicians for acceptability; and investigation into the existence of mechanisms to accredit, regulate and license HRH for quality. In line with the report, we measure AAAQ deficits by formulating four indices. Availability deficit (AvD) measures the deficit in the total stock of HRH, accessibility deficit (AsD) measures the maldistribution of rural HRH against their urban presence, acceptability deficit (ApD) measures the skew in cadre-mix and imbalance of female personnel relative to males within an HRH cadre (sex-mix), and quality deficit (QD) measures the deficit of qualified HRH.

AvD measures the deficit of available HRH against the contextually relevant requirement thresholds.

1 $${\mathrm{AvD}}_{\mathrm{cadre}} =1-\left(\frac{{P}_{\mathrm{cadre}}}{{R}_{\mathrm{cadre}}}\right)$$

AvD_cadre_ = availability deficit for a cadre, *P*_cadre_ = national density of available personnel for the cadre, *R*_cadre_ = requirement threshold for the cadre.

Cadrewise AvDs were calculated using available density (*P*) for 3 years 1981, 2001, and 2011 and the two requirement thresholds R-1 (Bhore) and R-2 (HLEG). Thus, six AvD values were calculated for each cadre. Theoretically, AvD can be positive, negative, or zero. When the density of available HRH is equal to the requirement threshold, AvD = zero, depicting no deficit or surplus. When the density of available HRH is less than the requirement threshold, AvD is positive depicting a deficit that needs attention. When the available HRH exceeds the requirement threshold, AvD is negative, representing a surplus.

AsD measures the deficit of HRH present in rural areas relative to their urban counterparts.

2 $${\mathrm{AsD}}_{\mathrm{cadre}}=1- \left(\frac{\frac{{P}_{\mathrm{Cadre}\_\mathrm{rural}}}{{R}_{\mathrm{cadre}\_\mathrm{rural}}}}{\frac{{P}_{\mathrm{cadre}\_\mathrm{urban}}}{{R}_{\mathrm{cadre}\_\mathrm{urban}}}}\right)=1- \left(\frac{{P}_{\mathrm{cadre}\_\mathrm{rural}}}{{P}_{\mathrm{cadre}\_\mathrm{urban}}}\right)$$

Here *R*_cadre_rural_ = *R*_cadre_urban_, AsD_cadre_ = accessibility deficit of a cadre, *P*_cadre_rural_ = density of available personnel for the given cadre in rural areas, *P*_cadre_urban_ = density of available personnel for the given cadre in urban areas, *R*_cadre_rural_ = requirement threshold for the cadre at the rural level, *R*_cadre_urban_ = requirement threshold for the cadre at the urban level. Here, we assumed that R-1 and R-2 for a given cadre to be the same for urban, rural, and total (national) groups, i.e., *R*_national_ = *R*_rural_ = *R*_urban_. This makes AsD independent of the requirement thresholds. Therefore, three AsD values for each cadre were calculated for corresponding to 3 years (1981, 2001, and 2011). Theoretically, AsD can be positive, negative, or zero. AsD becomes zero when the densities of available HRH in rural and urban areas are equal, i.e., equal accessibility in urban and rural areas. Positive AsD depicts a greater relative concentration of HRH in urban areas making rural access concerning. AsD is negative when rural HRH density surpasses its urban counterpart.

We present two ApD indices. First, ApD_cadre_mix_ measures the deficit of nursing and supporting cadres relative to doctors. Here, ANMs and nurses together constitute nursing cadres, while pharmacists and nursing cadres together form supporting cadres. GHWA explains—“the communities accept new intermediary professions such as medical assistants, surgery technicians, auxiliary nurses and lay health workers when some conditions are met” [10]. With this rationale, the report uses—cadre-mix of nurses and physicians as a proxy for acceptability and measures the nurses to physicians ratio to calculate the same. Along these lines, we have measured ApD_cadre-mix_ using the relative availability of supporting cadres (i.e., available density of supporting cadres /requirement threshold of supporting cadre) and the relative availability of doctors (i.e., available density of doctors/required density of doctors).

3 $${\mathrm{ApD}}_{\mathrm{cadre}\_\mathrm{mix}}=1-\left(\frac{\frac{{{P}_{\mathrm{cadre}}}}{{R}_{\mathrm{cadre}}}}{\frac{{P}_{\mathrm{doctor}}}{{R}_{\mathrm{doctor}}}}\right)$$

Here cadres are supporting and nursing cadres, ApD_cadre_mix_ = acceptability deficit of a cadre group, *P*_cadre_ = national density of available cadre groups, *P*_doctor_ = national density of doctors, *R*_cadre_ = requirement threshold for the cadre groups, *R*_doctor_ = REQUIREMENT threshold for doctors. Here, cadre groups refer to nursing and supporting cadres. ApD_cadre_mix_ was calculated for the nursing and supporting cadres for 3 years 1981, 2001, and 2011 using both R-1 and R-2. Six ApD_cadre_mix_ values were calculated for two cadre groups. Theoretically, ApD_cadre_mix_ can be positive, negative, or zero. When the relative availability of doctors is equal to the relative availability of the considered cadre group (nursing or supporting cadres), ApD_cadre_mix_ is zero, depicting acceptable cadre composition in the workforce. When the relative availability of cadre groups is smaller or greater than that of doctors, ApD_cadre_mix_ is positive or negative, respectively, denoting suboptimal acceptability that needs intervention. It is desirable to have ApD_cadre_mix_ value in the proximity of zero to ensure balanced cadre-mix and acceptability.

Second, we considered the within-cadre sex composition as a proxy for acceptability. The WHO–GHWA report proposes same-sex provider as one of the characteristics of acceptable HRH especially in populations, where being served by someone of the other sex is not culturally acceptable [10]. ApD_sex_mix_ measures the imbalance of female personnel relative to males for a cadre.

4 $${\mathrm{ApD}}_{\mathrm{sex}\_\mathrm{mix}} =1-\left(\frac{{P}_{\mathrm{cadre}\_\mathrm{female}}}{{P}_{\mathrm{cadre}\_\mathrm{male}}}\right)$$

ApD_sex_mix_ = Acceptability deficit of a cadre, *P*_cadre_female_ = national density of available females of an HRH cadre, *P*_cadre_male_ = national density of available males of the same cadre.

ApD_sex_mix_ is independent of requirement thresholds and depends only on the densities of available female and male personnel of an HRH cadre. Therefore, three ApD_sex_mix_ values for the years 1981, 2001, and 2011 were calculated for each cadre except ANMs who are supposed to be female health workers. Theoretically, ApD_sex_mix_ can be negative, positive, or zero. When female and male densities of cadre are equal, ApD_sex_mix_ is zero showing a sex-balanced acceptable workforce. Positive and negative ApD_sex_mix_ values denote skew towards males and females, respectively. ApD_sex_mix_ closer to zero is favorable for an acceptable workforce.

QD measures the deficit of qualified HRH relative to the total HRH, i.e., qualified and unqualified. Here, qualified HRH are those possessing educational qualifications proposed by the registration body or council for the cadre as given in Additional file 2: Table S3.

$${\mathrm{QD}}_{\mathrm{cadre}}=1-\left(\frac{\frac{{P}_{\mathrm{cadre}\_\mathrm{qualified}}}{{R}_{\mathrm{cadre}\_\mathrm{qualified}}}}{\frac{{P}_{\mathrm{cadre}}}{{R}_{\mathrm{cadre}}}}\right)=1-\left(\frac{{P}_{\mathrm{Cadre}\_\mathrm{qualified}}}{{P}_{\mathrm{cadre}}}\right)$$

Here *R*_cadre_qualified_ = *R*_cadre_, QD_cadre_ = quality deficit for a cadre, *P*_cadre_qualified_ = national density of qualified personnel in a given cadre, *P*_cadre_ = national density (qualified and unqualified) of the cadre, *R*_cadre_qualified_ = requirement threshold for qualified HRH in the cadre, *R*_cadre_ = requirement threshold for total HRH in the cadre.

We assume that the requirement thresholds are equal for qualified and total HRH. Hence, QD here is independent of requirement thresholds and is determined by the proportion of qualified HRH relative to total HRH. For each cadre, three QD values were calculated for 3 years (1981, 2001, and 2011). QD can range from 0 to 1. When all available personnel belonging to an HRH cadre are qualified, QD becomes zero, depicting the desired scenario of high HRH quality. The worst-case scenario with no qualified personnel is depicted by QD = 1. Since total HRH cannot be less than qualified HRH, QD cannot be negative.

### Data analysis

*Analysis 1:* We screened all NHPI reports for HRH-related recommendations to evaluate the incorporation of AAAQ dimensions and investigate the focussed cadres. Each recommendation was coded according to—addressed dimensions, targeted cadres, recommended strategies, and suggested/employed actions (Additional file 2: Tables 4a, b, c). Dimension, strategy, and action targeted by a policy recommendation were identified using the proposed framework (Fig. 1). Strategies and actions enlisted in the framework but not mentioned for any cadre in an NHPI for a given dimension were noted as ‘Strategy/Action not used’ (Table 2). Recommendations not specific to any cadres were coded as ‘non-cadre-specific’. Cadres apart from the six mentioned above were grouped as ‘Other’. For each NHPI report, we recorded the total number of dimensionwise and cadrewise recommendations independently and further cross-tabulated them. The policy focus was determined by the number of mentions. Variations in the total number of mentions across NHPIs were noted to determine changing focus. This approach has been previously used in various policy-content analyses [28, 29] that investigate policy focus or issue prioritization, including the Lancet Commission on Global Surgery [30].

**Table 2:** Cadrewise HRH-related strategies and actions for AAAQ dimensions recommended in the NHPIs

NHPI Year	Availability			Accessibility			Acceptability			Quality		
	Cadre focused	Recommendations given	Strategy/Action not used	Cadre focused	Recommendations given	Strategy/Action not used	Cadre focused	Recommendations given	Strategy/Action not used	Cadre focused	Recommendations given	Strategy/Action not used
1983	Other^a^	- Create a new HRH cadre	- Establish new training institutes - Increase the number of seats in existing training institutes - Task shifting and task sharing - Recruiting HRH from foreign countries - Retaining HRH within the country	Doctor	- Financial incentive	- Tele-consultation - Identify groups/individuals motivated to work in underserved areas - Remove professional isolation - Remove administrative barriers in recruitment like walk-in interviews - Mandatory rural postings	No recommendation focused on the acceptability of HRH			Other	- Formal training courses for unqualified HRH	- Grievance redressal and feedback system for patients - Maintain quality of HRH - Professional councils for all HRH cadres - Improving the training of HRH cadres - Standard licensing exam for all cadres - Conduct meetings to review common medical errors - Regular assessment of in-service staff
	Non-cadre-specific^b^	- Measure and monitor availability of HRH using information systems		AYUSH^c^	- Streamline and integrate traditional HRH cadres - Task shifting in underserved areas					Non-cadre- specific	- Changes in curriculum - Develop interpersonal/ soft skills	
				Non-cadre- specific	- Attract and retain HRH from surplus sector/area/level of care/system of medicine to underserved areas - Increase production of HRH in underserved areas - Develop information systems and tools to measure and monitor the availability of HRH							
2002	Doctor	- Develop information systems and tools to measure and monitor the availability of HRH - Establish new training institutes - Increase the number of seats in medical institutes - Task shifting and sharing	- Recruiting HRH from foreign countries - Retaining HRH within the country	Doctor	- Mandatory rural posting - Removing administrative barriers of recruitment - Task shifting and sharing in underserved areas - Tele-consultation	- Establish training institutes in underserved areas - Provide financial and non-financial incentives - Identify groups or individuals motivated towards serving underserved areas - Removing professional isolation - Develop information systems and tools to measure and monitor the geographical distribution of HRH - Streamline and integrate traditional medicine HRH in underserved areas	Non-cadre- specific	- Create and deploy HRH representative of sex, age, religion, etc. of the population being served	- Create HRH closer to the community - Deploy HRH in the local community - Induction training of new HRH - Create appropriate cadre-mix	Doctor	- Change the curriculum to suit all levels of care - Improve the training of HRH cadres - Continued medical education and training to HRH	- Regular assessment of in-service staff and performance-based incentives - Grievance redressal and feedback system for patients to identify areas of improvement for HRH - Standard licensing exam - Establish policy/rules for promotion, transfer, leave, salary, etc. for all HRH - Conduct meetings to review the common medical error - Formal training of unqualified HRH
	Nurse	- Increase the number of nursing institutes - Task-shifting and task-sharing - Develop information systems and tools to measure and monitor the availability of HRH		Nurse	- Task-shifting and multi-tasking of HRH cadres in underserved areas		Other	- Preferentially expand cadres with greater local acceptance		Non-cadre- specific	- Develop interpersonal/ soft skills in all cadres - Improving the training of HRH cadres	
	Other	- Establish new training institutes - Create a new HRH cadre (LMP^d^)		Paramedic (pharmacist)	- Task-shifting and multi-tasking of HRH cadres in underserved areas		Paramedic (pharmacist)	- Task-shifting and multi-tasking of HRH cadres		Paramedic (pharmacist)	- Changing the curriculum to suit all levels of care - Professional councils for all HRH cadres	
	Dentist	- Establish new training institutes		Other	- Create a new cadre (LMP) specifically for underserved areas		Doctor	- Develop socio-cultural competence in HRH		Nurse	- Improving the training of nurses	
	Paramedic (pharmacist)	- Task-shifting and multi-tasking		Non-cadre specific	- Task-shifting and multi-tasking of HRH cadres in underserved areas							
	Non-cadre specific	Create a new HRH cadre										
2017	Doctor	- Establish new training institutes - Increase the number of seats in existing institutes - Task shifting and sharing - Create a new HRH cadre	- Recruiting HRH from foreign countries - Reducing emigration - Reduce attrition	Doctor	- Establish training institutes in underserved areas - Mandatory rural posting - Remove administrative barriers in recruitments - Increase production in underserved areas - Tele-consultation - Providing financial and non-financial incentives - Identify individuals/groups motivated to work in underserved areas - Task shifting and multi-tasking of HRH cadres in underserved areas	- Reduce professional isolation - Develop information systems and tools to measure and monitor the geographical distribution of HRH	Doctor	- Emphasize socio-cultural aspects in the medical curriculum - Mandatory rural posting	- Create appropriate cadre-mix - Create and deploy HRH representative with the composition of society in terms of sex, caste religion, etc - Pre-posting regional training (induction training)	Doctor	- Improving the training of HRH cadres - Standard licensing exam for all cadres - Continued medical education and training to HRH - Develop interpersonal/ soft skills in HRH cadres - Changing the curriculum to suit all levels of care - Give performance-based incentives	- Regular assessment of in-service staff - Patient feedback and grievance redressal system - Conduct meetings to review common medical errors
	Nurse	- Create a new HRH cadre - Establish new training institutes					AYUSH	Expand cadres with high local acceptance preferentially				
	Other	- Task shifting and multi-tasking of HRH cadres - Create a new HRH cadre - Establish new training institutes - Task shifting and task sharing - Increase the number of seats in existing institutes - Develop information system tools to measure and monitor the availability of HRH		Paramedic (pharmacist)	- Task shifting and multitasking of HRH cadres in underserved areas - Increase production of HRH in underserved areas		Paramedic (pharmacist)	- Expand cadres with high local acceptance preferentially - Deploy HRH in the local community		Nurse	- Improving training of HRH cadres - Professional councils for all HRH cadres - Continued medical education and training to HRH	
	Paramedic (pharmacist)	- Increase the number of seats in existing training institutes, - Task shifting and task sharing, - Develop tools to measure HRH (by IPHS^e^ norms)		AYUSH	- Streamline and integrate traditional HRH in underserved areas - Tele-consultation - Task shifting and multi-tasking of HRH cadres in underserved areas		Nurse	- Expand cadres with high local acceptance preferentially		Other	- Improving training of HRH cadres - Formal training courses for unqualified HRH - Standard licensing exam - Professional councils - Develop interpersonal/ soft skills in HRH cadres	
	Non-cadre- specific	- Develop information system and tools to measure and monitor availability of HRH - Establish new training institutes - Increase number of seats in existing training institutes		Other	- Increase production of HRH in underserved areas - Task shifting and multi-tasking of HRH cadres in underserved areas - Remove administrative barriers in recruitment - Identify groups/individuals motivated to work in underserved areas - Create HRH cadre specifically for underserved areas		ANM	- Expand cadres with high local acceptance preferentially		Non-cadre- specific	- Continued medical education and training to HRH - Establish policy/ rules for promotion, transfer, leave, salary, etc. for all HRH cadres - Develop interpersonal/ soft skills in HRH cadres - Professional councils for all HRH cadres - Changing curriculum to suit all levels of care	
				ANM	- Task shifting and multi-tasking of HRH cadres in underserved areas		Other	- Create HRH closer to community -Expand cadres with high local acceptance preferentially - Task shifting and multi-tasking of HRH cadres		AYUSH	- Changing curriculum to suit all levels of care - Professional councils	
				Nurse	- Task shifting and multi-tasking of HRH cadres in underserved areas - Create new HRH cadre specifically for underserved areas					Paramedics (pharmacist)	- Changing curriculum to suit all levels of care - Professional councils	
				Non-cadre-specific	- Provide financial and non-financial incentives					Dentist	- Professional councils for dentist	

*HRH* Human Resources for Health, *NHPI* National Health Policy of India, *AAAQ* availability, accessibility, acceptability, quality, ^a^Other includes mid-level practitioners, community health workers, and multi-purpose workers. ^b^Non-cadre-specific recommendations apply to all HRH cadres. ^c^AYUSH: Ayurveda, Yoga, and Naturopathy, Unani, Siddha and Homeopathy, ^d^LMPs: Licentiate Medical Practitioners, ^e^IPHS: Indian Public Health Standards

*Analysis 2:* We calculated cadrewise AAAQ deficit indices for pre-NHPI census years (1981, 2001, 2011). For each year, we obtained two AvD and ApD values (using R-1 and R-2) and one AsD and QD value (as they are independent of R) for each cadre. Index values were categorized as critical (1 to 0.75), high (0.74 to 0.50), moderate (0.49 to 0.25), and low (0.24 to 0) deficit. The surplus was categorized as low (− 0.01 to − 0.24), moderate (− 0.25 to − 0.49), high − 0.50 to − 0.74), and extreme (< − 0.75). We conducted two sub-analyses:

(a) We looked at the time trends in the AAAQ deficits from 1981 to 2011. For each dimension, we evaluated longitudinal change in deficit indices values for each cadre between 1981 and 2011 by measuring percent change using the following equation.

6 $$\mathrm{Percentage change in }D= \left(\frac{{D}_{2011}-{D}_{1981}}{{D}_{1981}}\right)X 100$$

*D* = deficit index, i.e., AvD, AsD, ApD, or QD. *D*_2011_ = deficit index for 2011, *D*_1981_ = deficit index for 1981.

For availability and acceptability, since two sets of cadrewise deficit indices were calculated for each year (using R1 and R2), time trends were observed separately for indices based on each requirement threshold.

(b) To look into the statuses of availability, accessibility, acceptability, and quality of the cadres before each NHPI, we conducted retrospective situational analyses using the deficit index values of pre-NHPI census years. It was essential to use the deficit indices based on then-existing (relevant) requirement thresholds. R-1 (Bhore requirement thresholds—1946) is relevant for NHPI-1983 and NHPI-2002, and R-2 (HLEG requirement thresholds—2011) is relevant for NHPI-2017. AvD and ApD calculated using P-1981 (present HRH density for 1981) and P-2001 against R-1 were used for situational analysis for NHPI-1983 and 2002, respectively, while AvD and ApD calculated using P-2011 and R-2 were used for NHPI-2017. Considerations for requirement thresholds were not needed for situational analyses of AsD and QD as these indices are independent of thresholds.

*Analysis 3:* To investigate policy match or mismatch, i.e., whether NHPI recommendations targeted the deficient HRH dimensions and cadres, we compared focused cadres (i.e., most mentioned cadres) for a given dimension in all NHPIs with cadres’ deficit/surplus categories (low, moderate, high, critical) for preceding census years. For instance, we looked at if cadres with high deficits had more focus in the NHPI recommendations.

## Results

The section numbers correspond to the three analyses described in the “Methods”.

1. Dimensionwise and cadrewise distribution of NHPI recommendations

**Fig. 2:**
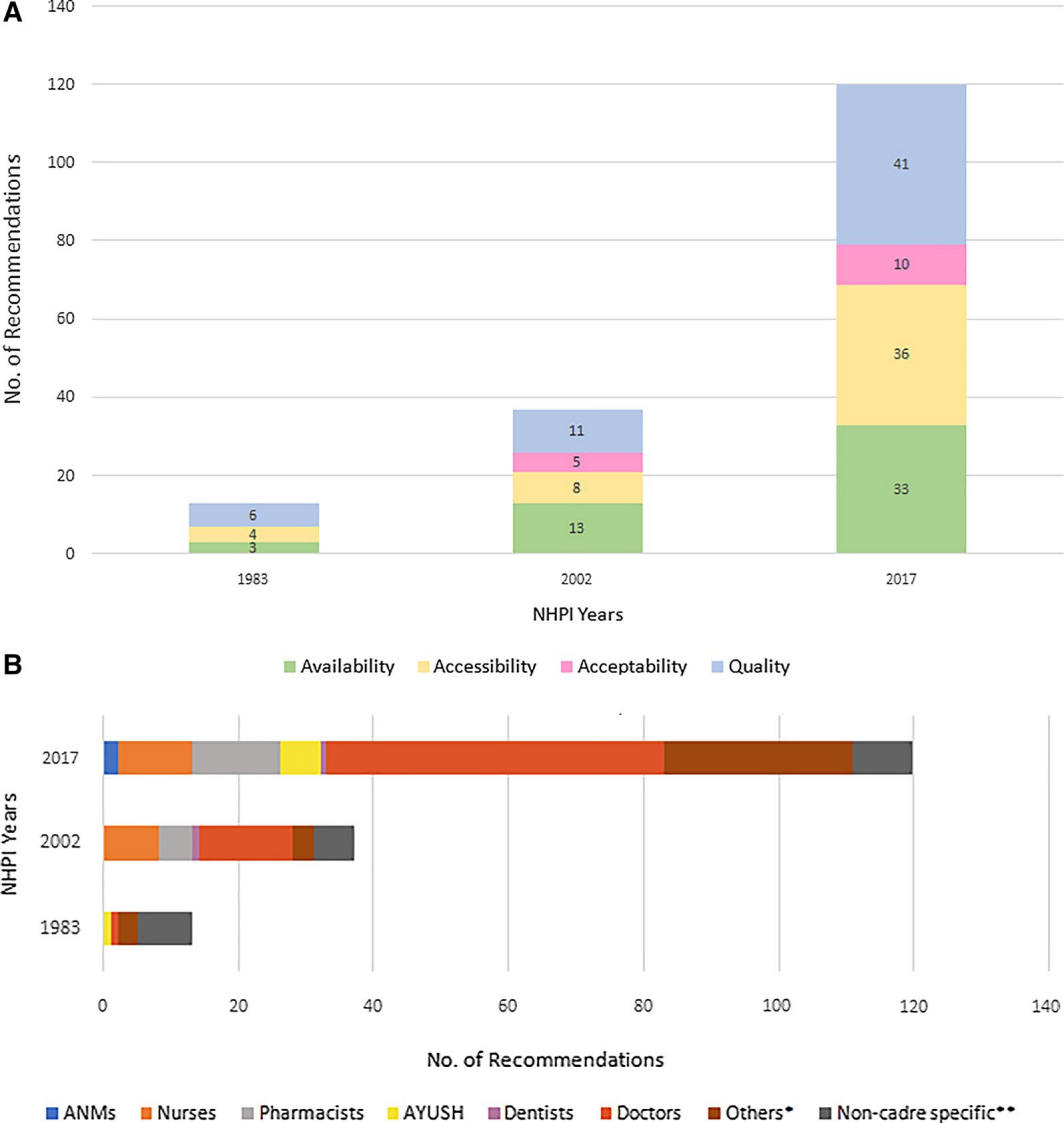
**A** Dimensionwise distribution of HRH-related recommendations of NHPI—1983, 2002, and 2017. Numbers written inside the bars denote the number of recommendations. *HRH* Human Resources for Health, *NHPI* National Health Policy of India. **B** Cadrewise distribution of HRH-related recommendations of NHPI 1983, 2002, 2017. *Other includes mid-level practitioners, community health workers, and multi-purpose workers. **Non-cadre-specific recommendations apply to all HRH cadres. *HRH* Human Resources for Health, *NHPI* National Health Policy of India, *HRH* Human Resources for Health, *AYUSH* Ayurveda, Yoga and Naturopathy, Unani, Siddha and Homeopathy, *ANM* auxiliary nurse-midwife

The distribution of recommendations according to AAAQ dimensions for three NHPIs is shown in Fig. 2A. Over years, the dimensional focus shifted from HRH quality to availability and back to quality. The focus on accessibility and acceptability gradually increased across NHPIs, with a greater improvement for accessibility than acceptability. The commonly proposed strategies for improving availability, accessibility, acceptability, and quality were: establishing new and expanding existing training institutes, task shifting/sharing, preferential expansion of cadres with high local acceptance, and need-based changes in training, respectively (Table 2). Retaining HRH within the country, reducing professional isolation, pre-posting induction training, and regular assessment of in-service staff were the least commonly prescribed strategies/actions for availability, accessibility, acceptability, and quality, respectively, across NHPIs (Table 2). The cadrewise distribution of recommendations across NHPIs is shown in Fig. 2B. Doctors were prioritized across all NHPIs with the highest recommendations, while ANMs and dentists had the least recommendations. The commonly proposed AAAQ strategies/actions for doctors were—increasing the training capacity of institutes, imposing mandatory rural service, developing socio-cultural competence, and incorporating changes in the curriculum. Improvement in training (quality-related recommendation), recruiting AYUSH in underserved areas (accessibility), and establishing professional councils (quality) were common strategies for nurses, AYUSH, and paramedics, respectively (Table 2). Cross-tabulation of cadrewise and dimensionwise number of recommendations is given in Table 3.

2. a. Longitudinal trends of dimensionwise deficits over three decades

**Table 3:** Number of cadrewise recommendations of HRH in the NHPIs for AAAQ dimensions

Cadres	Dimensionwise recommendations											
	Availability			Accessibility			Acceptability			Quality		
	1983	2002	2017	1983	2002	2017	1983	2002	2017	1983	2002	2017
Doctor	0	5	15	1	4	15	0	1	2	0	4	18
Nurse	0	4	2	0	1	2	0	0	1	0	3	6
AYUSH^a^	0	0	0	1	0	4	0	0	1	0	0	1
ANM^b^	0	0	0	0	0	1	0	0	1	0	0	0
Dentist	0	1	0	0	0	0	0	0	0	0	0	1
Pharmacist	0	1	5	0	1	3	0	1	2	0	2	3
Other^c^	1	1	10	0	1	9	0	1	3	2	0	6
Non-cadre-specific^d^	2	1	1	2	1	2	0	2	0	4	2	6
Total recommendations	3	13	33	4	8	36	0	5	10	6	11	41

*HRH* Human Resources for Health, *NHPI* National Health Policy of India, *AAAQ* Availability, Accessibility, Acceptability, ^a^AYUSH: Ayurveda, Yoga and Naturopathy, Unani, Siddha and Homeopathy, ^b^ANM: Auxiliary Nurse-Midwife, ^c^Other includes mid-level practitioners, community health workers, and multi-purpose workers. ^d^Non-cadre-specific recommendations apply to all HRH cadres

**Fig. 3:**
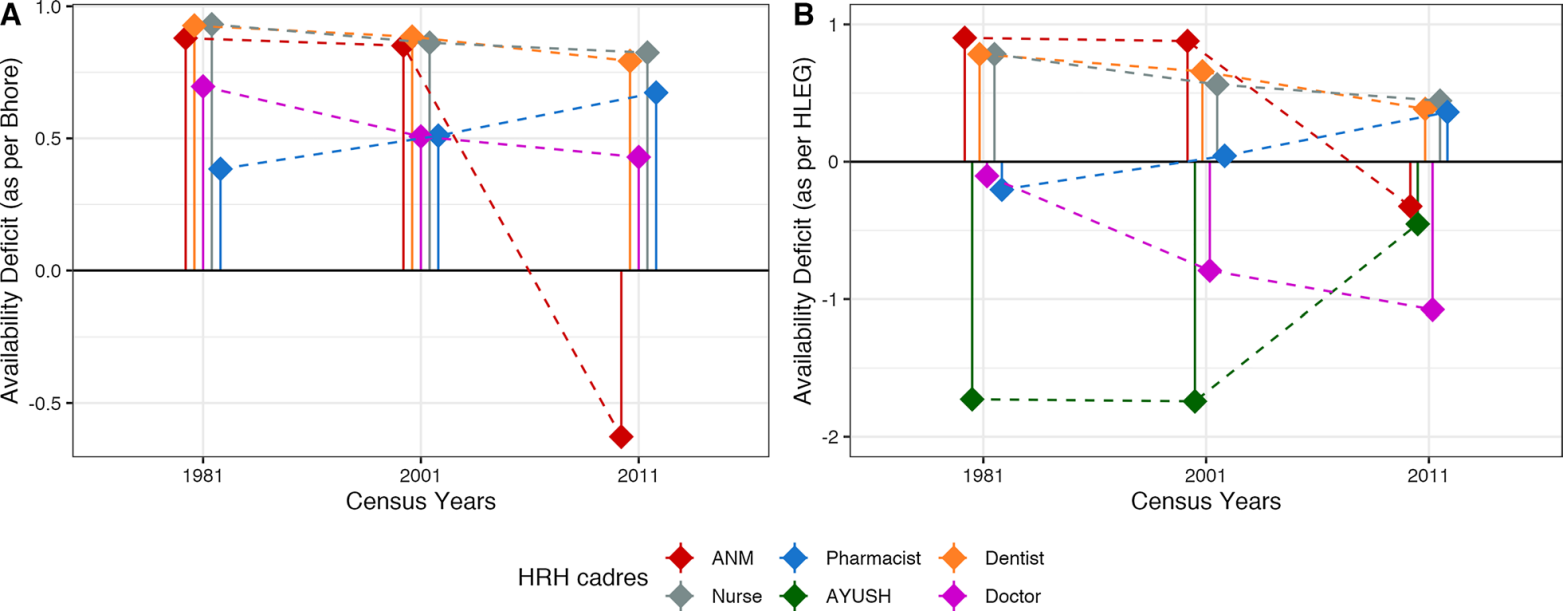
Availability Deficit (AvD) for pre-NHPI census years 1981, 2001, 2011 according to Bhore and HLEG thresholds. Dashed lines indicate the longitudinal changes in deficit. Solid vertical lines indicate availability deficits. Availability deficits for AYUSH were not calculated using Bhore Committee Report due to the unavailable requirement threshold. *HRH* Human Resources for Health, *NHPI* National Health Policy of India, *HLEG* High-Level Expert Group, *AYUSH* Ayurveda, Yoga and Naturopathy, Unani, Siddha and Homeopathy, *ANM* auxiliary nurse-midwife

**Fig. 4:**
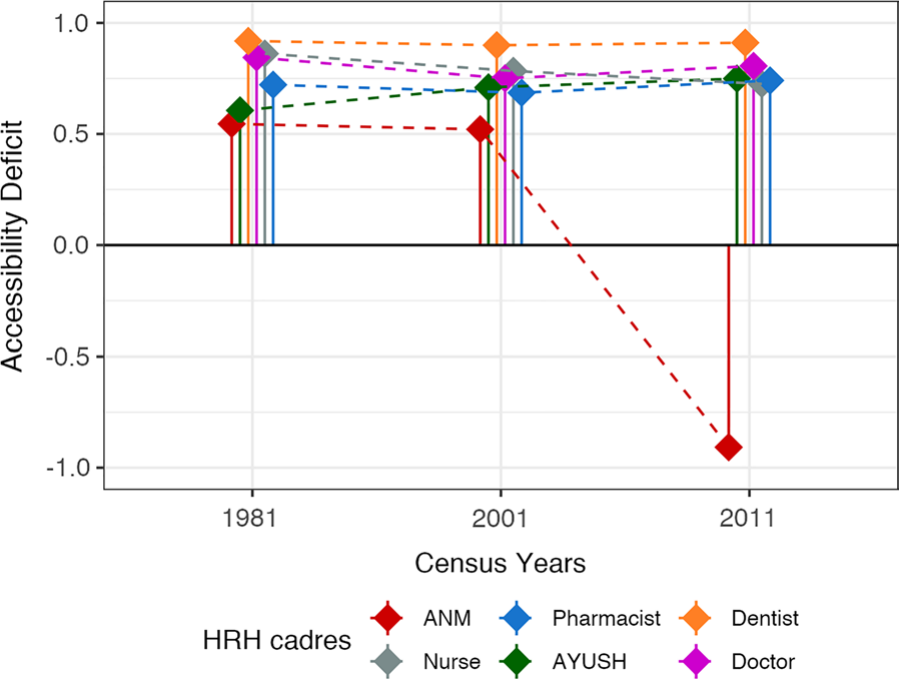
Accessibility Deficit (AsD) for pre-NHPI census years 1981, 2001, 2011. Dashed lines indicate the longitudinal changes in deficit. Solid vertical lines indicate Accessibility Deficit. *HRH* Human Resources for Health, *NHPI* National Health Policy of India, *AYUSH* Ayurveda, Yoga and Naturopathy, Unani, Siddha and Homeopathy, *ANM* auxiliary nurse-midwife

**Fig. 5:**
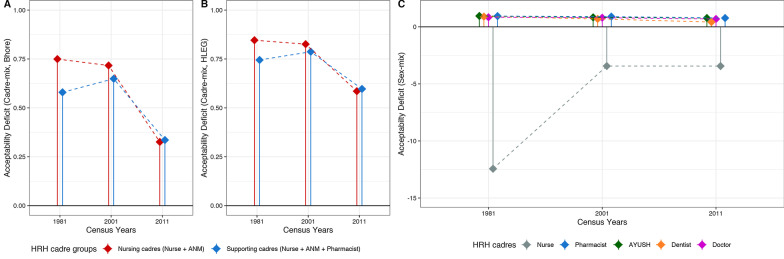
Acceptability Deficit (ApD) for pre-NHPI census years 1981, 2001, 2011 according to Bhore and HLEG thresholds. Dashed lines indicate the longitudinal changes in acceptability deficit. Solid vertical lines indicate an acceptability deficit. Nursing cadres include ANM and nurses. Supporting cadres include pharmacists along with nursing cadres. ApD (sex-mix) was not calculated for ANMs as an ANM by definition is a female health worker. *HRH* Human Resources for Health, *NHPI* National Health Policy of India, *HLEG* High-Level Expert Group, *ANM* auxiliary nurse-midwife

**Fig. 6:**
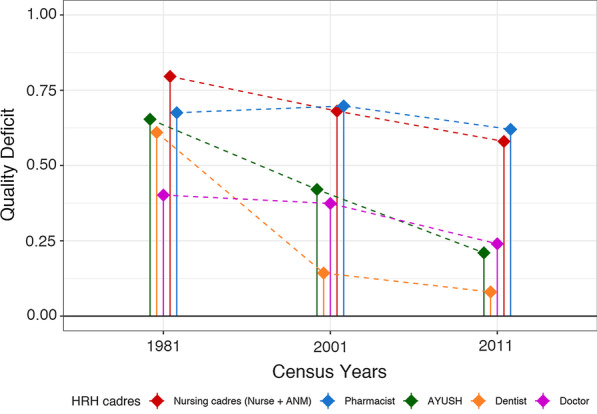
Quality deficit (QD) for pre-NHPI census years 1981, 2001, 2011. Dashed lines indicate the longitudinal changes in quality deficit. Solid vertical lines indicate quality deficit. Combined quality deficits for nurses and ANMs were calculated as proportions of qualified HRH were not available for these cadres separately. *HRH* Human Resources for Health, *NHPI* National Health Policy of India, *AYUSH* Ayurveda, Yoga and Naturopathy, Unani, Siddha and Homeopathy, *ANM* auxiliary nurse-midwife

National AvD calculated using both thresholds showed a decreasing trend for all cadres from 1981 to 2011, except for pharmacists and AYUSH for whom the deficit increased. The steepest decrease was seen for ANMs as per Bhore (171%) (Fig. 3A) and HLEG (136%) thresholds (Fig. 3B). AvD increased for pharmacists by 75% using the Bhore threshold (R-1) and for AYUSH by 74% as per the HLEG threshold (R-2). AsD was almost constant (< 1% change) from 1981 to 2011 for all cadres. However, for ANMs, AsD decreased steeply between 2001 and 2011 (Fig. 4). ApD_cadre-mix_ showed a decreasing trend under both requirement thresholds for nursing and supporting cadres (Fig. 5A, B). ApD_sex-mix_ showed a little decrease (< 15%) for pharmacists, AYUSH, and doctors, and a 53% decrease for dentists. Acceptability for nurses also improved as witnessed by ApD_sex-mix_ approaching zero from 1981 to 2011, indicating an increase in male nurses. QD for all cadres declined with the fall being steepest for dentists (87%) and least for pharmacists (8%) (Fig. 6). Thus, all deficit indices showed a decreasing trend of variable magnitudes from 1981 to 2011 except AsD, denoting improvements in HRH availability, acceptability, and quality; however, the accessibility of HRH in rural areas has failed to improve for several cadres.

- 2. b.Retrospective situational analyses of HRH for pre-NHPI years

In 1981, all cadres had critical-to-moderate AvD with the greatest deficit for nurses (Fig. 3A). AsD for all cadres was either critical or high, with dentists having the greatest accessibility deficit (Fig. 4). Critical and high ApD_cadre-mix_ were noted for nursing and supporting cadres, respectively (Fig. 5A). Critical ApD_sex-mix_ were noted for all cadres except nurses (Fig. 5C). QDs ranged from critical to moderate with nursing cadres (ANMs and nurses) presenting the highest quality deficit (Fig. 6). In 2001, all cadres had critical to high AvDs with dentists being the most deficiently available (Fig. 3A). AsD for all cadres was critical with the greatest accessibility deficit for dentists and least for ANMs (Fig. 4). High ApD_cadre-mix_ was noted for both nursing and supporting cadres (Fig. 5A). ApD_sex-mix_ was critical for all cadres except nurses (Fig. 5C). QD was high or moderate for all cadres except for dentists that showed a low value. (Fig. 6). In 2011, pharmacists, nurses, and dentists had moderate AvD, and ANMs, AYUSH, and doctors had moderate, high, and extreme surpluses, respectively (Fig. 3B). All cadres showed high and critical AsDs except for ANMs which presented a high surplus for accessibility (Fig. 4). Nursing and supporting cadres both had high ApD_cadre-mix_ (Fig. 5B). ApD_sex-mix_ was high or critical for all cadres except nurses (Fig. 5C). QD was critical for pharmacists and low for dentists (Fig. 6). Thus, the situational analyses for the three pre-NHPI census years point out that before each NHPI all dimensions for all cadres were in a deficit with only two exceptions. Availability of AYUSH, dentists, and doctors and accessibility of ANMs were in surplus before NHPI 2017.

3. Comparison of cadrewise deficit categories with cadres focused in NHPIs

Table 4 shows that no cadre-specific recommendations were made for availability in NHPI-1983 although the majority of cadres suffered critical to high AvD. Most cadres showed critical to high AsD; however, only one recommendation was made for doctors and AYUSH each. There were no acceptability-related recommendations though all cadres had high or critical ApDs. No recommendations were made to improve the HRH quality although the majority of cadres had critical or high QDs. In NHPI-2002, no availability-related recommendations were made for ANMs and dentists that had critical AvDs. Dentists and nurses with critical AsDs had zero and one accessibility-related recommendation, respectively, while AYUSH and ANMs with high AsD had none. Only one acceptability-related recommendation was made for doctor and pharmacist each, while all cadres had high or critical ApDs. Pharmacists with high QDs had only two quality-related recommendations, while doctors with moderate QD had four recommendations. In NHPI-2017, there were 15 availability-related recommendations for doctors who had extreme availability surplus, while dentists with moderate AvD had no recommendations. Nurses and pharmacists with high AsD had two and three recommendation(s), respectively, while dentists with critical deficits had none. One acceptability-related recommendation was made for AYUSH and nurses each that showed critical ApD and extreme acceptability surplus, respectively. Doctors had 18 quality-related recommendations despite low QD. Thus, the recommendations in NHPIs did not correspond to the situational deficits in many instances indicating a mismatch with the policy focus.

**Table 4:** Number of cadrewise NHPI recommendations compared with their deficit categories for AAAQ dimensions

	1983			2002			2017		
Dimension	Cadre	Number of Recommendations	Deficit category using Bhore threshold	Cadre	Number of Recommendations	Deficit category using Bhore threshold	Cadre	Number of Recommendations	Deficit category using HLEG threshold
Availability	ANM^a^	0/3	Critical deficit	Doctor	5/13	High deficit	Doctor	15/33	Extreme surplus
	Nurse	0/3	Critical deficit	Nurse	4/13	Critical deficit	Pharmacist	5/33	Moderate deficit
	Dentist	0/3	Critical deficit	Pharmacist	1/13	High deficit	Nurse	2/33	Moderate deficit
	Doctor	0/3	High deficit	AYUSH	1/13	–	Dentist	0/33	Moderate deficit
	Pharmacist	0/3	Moderate deficit	Dentist	0/13	Critical deficit	ANM	0/33	Moderate surplus
	AYUSH^c^	0/3	–	ANM	0/13	Critical deficit	AYUSH	0/33	Moderate surplus
Accessibility	Doctor	1/4	Critical deficit	Doctor	4/8	Critical deficit	Doctor	15/36	Critical deficit
	AYUSH	1/4	High deficit	Nurse	1/8	Critical deficit	AYUSH	4/36	Critical deficit
	Nurse	0/4	Critical deficit	Pharmacist	1/8	High deficit	Pharmacist	3/36	High deficit
	Dentist	0/4	Critical deficit	Dentist	0/8	Critical deficit	Nurse	2/36	High deficit
	Pharmacist	0/4	High deficit	AYUSH	0/8	High deficit	ANM	1/36	Extreme surplus
	ANM	0/4	High deficit	ANM	0/8	High deficit	Dentist	0/36	Critical deficit
Acceptability (cadre-mix)	Nursing cadres ^d^	No acceptability-related recommendations	Critical deficit	Supporting cadres	1/5	High deficit	Supporting cadres	4/10	High deficit
	Supporting cadres ^e^		High deficit	Nursing cadres	0/5	High deficit	Nursing cadres	2/10	High deficit
Acceptability (sex-mix)	Doctor	No acceptability-related recommendations	Critical deficit	Doctor	1/5	Critical deficit	Pharmacist	2/10	Critical deficit
	Pharmacist		Critical deficit	Pharmacist	1/5	Critical deficit	Doctor	2/10	High deficit
	AYUSH		Critical deficit	AYUSH	0/5	Critical deficit	AYUSH	1/10	Critical deficit
	Dentist		Critical deficit	Dentist	0/5	High deficit	Nurse	1/10	Extreme surplus
	Nurse		Extreme surplus	Nurse	0/5	Extreme surplus	Dentist	0/10	Moderate deficit
Quality	Nursing cadres	0/6	Critical deficit	Doctor	4/11	Moderate deficit	Doctor	18/41	Low deficit
	Pharmacist	0/6	High deficit	Nursing cadres	3/11	High deficit	Nursing cadres	6/41	High deficit
	AYUSH	0/6	High deficit	Pharmacist	2/11	High deficit	Pharmacist	3/41	High deficit
	Doctor	0/6	High deficit	AYUSH	0/11	Moderate deficit	AYUSH	1/41	Low deficit
	Dentist	0/6	Moderate deficit	Dentist	0/11	Low deficit	Dentist	1/41	Low deficit

‘–’ indicates uncalculated deficit indices due to unavailable data. AvDs for AYUSH for the years 1981 and 2001 were not calculated due to the lack of requirement thresholds in the Bhore Committee Report. HRH deficit quartiles were classified as—critical (1 to 0.75), high (0.74 to 0.50), moderate (0.49 to 0.25), and low (0.24 to 0). The surplus was categorized as low (− 0.01 to − 0.24), moderate (− 0.25 to − 0.49), high (− 0.50 to − 0.74), and extreme (< − 0.75). *AAAQ* availability, accessibility, acceptability, ^a^ANM: Auxiliary Nurse-Midwife, ^c^AYUSH: Ayurveda, Yoga and Naturopathy, Unani, Siddha and Homeopathy, ^d^Nursing cadres include ANMs and nurses, ^e^Supporting cadres include nursing cadres and pharmacists

## Discussion

Our results show that NHPIs’ HRH-related recommendations do not systematically incorporate AAAQ dimensions. Over three decades, the main focus has been on HRH availability and quality with limited attention to accessibility and acceptability. Recommendations consistently seem to prioritize doctors over other cadres, such as ANMs and pharmacists. The situational analysis revealed dimensionwise deficits in most cadres for the years corresponding to all NHPIs. NHPIs did not always focus on the deficient cadres indicating policy priority mismatches and thus failing to fully address the situational HRH deficiencies. Longitudinally, the magnitude of deficits declined for all dimensions except accessibility showing that urban–rural HRH maldistribution is a chronic problem for the country.

Our analysis is significant in two ways. First, past studies have focused largely on HRH availability but not other dimensions. HRH availability in India [7] and other countries have been studied cross-sectionally [31] and longitudinally [32, 33], using density (HRH per unit population, e.g., HRH per 100,000) as a metric. Another widely used metric for measuring HRH availability is vacancy (required HRH—present HRH) [34]. Furthermore, their interpretations are heterogeneous, i.e., density is a positive measure (higher value of density is considered better), while vacancy is a negative measure (greater being worse). Density is relative to the population, while vacancy is relative to some requirement threshold. In this study, we propose a family of deficit indices that help to uniformly investigate all the four HRH AAAQ dimensions. All the indices have a similar interpretation, i.e., zero depicts no deficit, while the positive values depict deficiencies in dimensions that need policy attention. Population-based or threshold-based values are easily incorporated in the index calculations. Second, we use a framework-based approach for policy document analysis that allows evaluation against quantitative indices thereby improving analytical rigor and transparency of underlying assumptions. It can be used to monitor the progress of the National Health Mission (India) and translated to evaluate the HRH policies of other countries. In previous studies, HRH-related policies have been assessed through interview methods [33], process tracing [31], or qualitative summarization [35]. The proposed framework focuses on standardized policy analysis removing stakeholder bias seen in interviews and the investigator’s bias in qualitative summaries. The conjoint application of framework and indices generates information useful for rigorous process tracing.

Our analysis of NHPI recommendations fills an important gap as the 2014 WHO–GHWA report indicated that there was ‘insufficient data’ for reliance on India's HRH policy on the AAAQ dimensions [10]. In addition, the deficit analysis confirms previous findings for the deficiencies in HRH dimensions, such as shortages in HRH availability [36], skewed urban–rural distribution depicting disparities in accessibility [36], poor quality indicated by a substantial proportion of unregistered/unqualified HRH [36], and low nurse-to-physician ratios compromising workforce acceptability [10]. These findings speak to the importance of viewing HRH holistically through AAAQ dimensions.

Focusing on all four dimensions is crucial, because population health outcomes can improve only when high-quality HRH are available equitably in an acceptable form to the people [11]. In the past, policies focusing on increasing HRH production have had success in enhancing availability, but they have failed to improve the accessibility and quality of the workforce [37]. Moreover, acceptability and regulation of outsized unqualified HRH remain largely unaddressed [10, 38]. This can be corrected by integrating AAAQ dimensions in health policies [31]. Adopting and implementing policies that address these dimensions is crucial and demands several prerequisites—(a) identification of deficiencies in cadres through dimensionwise indices [32, 33], (b) identification of dimensionwise and cadrewise focus of HRH policies using a predetermined framework [35, 39], (c) assessing the mismatches between dimensional deficiencies in HRH and policy focus, (d) experimentation with and evaluation of strategies and actions for their relevance, effectiveness, and cost-effectiveness [37, 40].

There is an urgent need for a dedicated national HRH policy that measures needs, and develops, executes, and monitors plans to enhance the AAAQ of HRH in India. However, in the absence of a standalone HRH policy, restructuring Indian national and subnational policies to explicitly incorporate specific strategies and actions addressing AAAQ dimensions will allow for an evidence-based, coordinated, and sustained response towards solving the HRH crisis [10]. Cadre-specific policy recommendations should focus more on primary-care level cadres, such as ANMs, community health workers, nurses, and rural physicians. Beyond recommendations, policies should also discuss implementation strategies, such as reducing attrition in HRH, removing administrative barriers for deployment, developing socio-cultural competence, and regular assessment of in-service staff [41]. Robust efforts to continually develop and check HRH requirement thresholds are necessary to address the relevance of AAAQ indices. Currently, the NITI Aayog has set a target of 45 physicians, nurses, and midwives per 10,000 people to be achieved by 2030 under the SDGs program [42]. However, this focuses solely on HRH availability. Future policies should set targets for multiple indicators measuring AAAQ dimensions for comprehensive progress monitoring.

One of the strengths of this study is the introduction and application of a novel framework for the systematic analysis of HRH AAAQ dimensions in health policies and plans. Another novelty is the proposal of a family of indices that can measure availability, accessibility, different forms of acceptability, and quality of HRH with uniform and policy-relevant interpretation. However, the study has several limitations. First, data for qualification could not be derived from the census due to the lack of educational details for the self-reported occupations. Therefore, we applied the proportion of qualified HRH derived from NSS to relevant census years to obtain the absolute number of qualified HRH. While this can create some comparability challenges, a similar approach has been used in another study [24] and the comparability of NSS and census data has been discussed elsewhere [24]. Second, the availability deficit (AvD) could not be calculated for AYUSH practitioners using the requirement threshold based on Bhore Committee (R-1), since this cadre was not recognized back in 1946. Third, we could not calculate quality deficit (QD) for nurses and ANMs individually due to the resolution of the intersection of NOC codes with educational details in the NSS data. We, however, calculated the combined QD for nurses and ANMs. Fourth, following the approach of the Lancet Commission on Global Surgery [30] and that of other studies [28, 29], for policy content analysis, focus on AAAQ dimensions and HRH cadres in NHPIs was determined using the number of mentions. We understand that our approach is insufficient and does not consider aspects, such as the relevance, effectiveness, and cost-effectiveness of the policy recommendations [40]. However, the required data on additional aspects are usually absent in LMICs, and measuring the strength of strategies and actions was out of the scope of the study. Finally, here we assess HRH sex-mix and the cadre-mix as proxies of acceptability. We chose these proxies due to their inclusion in the WHO–GHWA report. However, there are other attributes that can be critical towards determining acceptability. In India’s context, these include age, religious, and caste composition of HRH, place of origin of the health workers, and their educational or training setting among other factors [43]. The ApD values calculated here might vary depending on the proxy of choice portraying that acceptability of HRH is composed of several attributes.

Future research can add to the study findings and improve upon its limitations in several ways. First, future studies can extend the ApD calculation to other attributes, such as age, religion, etc. It would also be important to synthesize a composite measure of ApD that can combine the multiple attributes in a non-compensatory or partially compensatory weighted average manner [44]. Second, validating the appropriateness of the thresholds used for AAAQ deficit calculations is also an important direction of work. Ideally, these thresholds should emerge from the consensus of the multiple stakeholders using a policy Delphi-method study [45]. Third, investigating and comparing the effectiveness and cost-effectiveness of the strategies and actions proposed in the current framework through observational and intervention studies could greatly benefit the larger field of health systems and policy research. Fourth, applying the framework and deficit indices to assess HRH policies and status at local levels (e.g., states/provinces, districts, municipalities etc.) would be useful for decentralized and targeted policymaking. Finally, the application of the integrated indices–framework approach in other low- and middle-income countries (LMICs) facing HRH problems [46] will allow cross-national comparison of strengths and weaknesses of HRH policies for contextual needs and generate evidence for strategies and actions instrumental for dimensionwise HRH improvements.

## Conclusions

NHPI recommendations addressing the HRH AAAQ dimensions need to be based on situational analysis of needs and informed by evidence. Furthermore, while availability and quality are focused explicitly in policies, adequate policy attention is not awarded to acceptability and accessibility that are critical for a well-functioning and equitable workforce. Formulating a dedicated HRH policy addressing the contextual HRH deficiencies is crucial for India. The framework and indices-based method can help identify the gaps between targeted and needed dimensions and cadres for effective HRH strengthening in countries. At the global level, the application of framework and indices will allow a comparison of the strengths and weaknesses of HRH-related policies and indicate implementation strategies and actions.

## Supplementary Information

**Additional file 1. **1.a. References for HRH strategies and actions in the AAAQ framework. Legend—HRH: Human Resources for Health, AAAQ: Availability, Accessibility, Acceptability, Quality. 1.b. Methods Supplement. Description of data: The file contains equations for calculations of R-1 (Bhore thresholds) and R-2 (HLEG thresholds) at health center levels and P for pre-NHPI census—1981, 2001, and 2011 years along with considerations and limitations used for calculations.

**Additional file 2: Table S1.** HRH categories with corresponding National Occupation Classification codes (NOC). Legend—HRH: Human Resources for Health, NOC: National Occupation Classification, ANM: Auxiliary Nurse-Midwife, AYUSH: Ayurveda, Yoga and Naturopathy, Unani, Siddha and Homeopathy. **Table S2.** Data tables used for extraction of HRH data from Census 1981, 2001, 2011. Legend—HRH: Human Resources for Health. **Table S3.** Qualifications of qualified HRH. Legend—HRH: Human Resources for Health, ANM: Auxiliary Nurse-Midwife, AYUSH: Ayurveda, Yoga and Naturopathy, Unani, Siddha and Homeopathy. **Table S4a.** Coding of HRH-related dimensions of NHPI 1983 into dimension, cadre, action, and strategy using the framework for HRH strengthening. Legend—NHPI: National Health Policy of India, HRH: Human Resources for Health, AYUSH: Ayurveda, Yoga and Naturopathy, Unani, Siddha and Homeopathy, CHW: Community Health Worker. Other includes mid-level practitioners, community health workers, and multi-purpose workers. Non-cadre-specific recommendations are the ones that are not specific to any cadre. **Table S4b.** Coding of HRH-related dimensions of NHPI-2002 into dimension, cadre, action, and strategy using the framework for HRH strengthening. Legend—NHPI: National Health Policy of India, HRH: Human Resources for Health, AYUSH: Ayurveda, Yoga and Naturopathy, Unani, Siddha and Homeopathy, CHW: Community Health Worker. Other includes mid-level practitioners, community health workers, and multi-purpose workers. Non-cadre-specific recommendations are the ones that are not specific to any cadre. **Table S4c.** Coding of HRH-related dimensions of NHPI-2017 into dimension, cadre, action, and strategy using the framework for HRH strengthening. Legend—NHPI: National Health Policy of India, HRH: Human Resources for Health, AYUSH: Ayurveda, Yoga and Naturopathy, Unani, Siddha and Homeopathy, CHW: Community Health Worker. Other includes mid-level practitioners, community health workers, and multi-purpose workers. Non-cadre-specific recommendations are the ones that are not specific to any cadre.

## Data Availability

The data sets supporting the conclusions of this article are included with the article and its additional files.

## Abbreviations

HRH: Human Resources for Health
AAAQ: Availability, accessibility, acceptability, and quality
NHPI: National Health Policy of India
UHC: Universal Healthcare Coverage
HLEG: High-level Expert Group
GHWA: Global Health Workforce Alliance
AvD: Availability deficit
AsD: Accessibility deficit
ApD: Acceptability deficit
QD: Quality deficit
ANMs: Auxiliary nurse-midwives
AYUSH: Ayurveda, Yoga and Naturopathy, Unani, Siddha and Homeopathy
LMICs: Low- and middle-income countries
CHW: Community health worker
LMP: Licentiate medical practitioner
ASHA: Accredited Social Health Activist
NOC: National Occupation Classification
NSS: National Sample Survey
MoHFW: Ministry of Health and Family Welfare
WHO: World Health Organization

## References

[1] Guilbert JJ (2006). The World Health Report 2006: working together for health. Educ Health (Abingdon).

[2] Speybroeck N, Kinfu Y, Dal Poz MR, Evans DB. Reassessing the relationship between human resources for health, intervention coverage and health outcomes. WHO, Geneva: World Health Organization (WHO); 2006 Mar.

[3] United Nations Development Programme. Human resources for health | United Nations Development Programme. https://www.undp-capacitydevelopment-health.org/en/capacities/focus/programme-management/human-resources/. Accessed 19 Aug 2021.

[4] World Health Organization (WHO). Framing the health workforce agenda for the Sustainable Development Goals: Biennium report 2016–2017 WHO health workforce. World Health Organization (WHO); 2017.

[5] World Health Organization (WHO). Global strategy on human resources for health: Workforce 2030. World Health Organization (WHO); 2016.

[6] BMJ Newsroom. Skilled health workforce in India does not meet WHO recommended threshold | BMJ . 2019. https://www.bmj.com/company/newsroom/skilled-health-workforce-in-india-does-not-meet-who-recommended-threshold/. Accessed 19 Aug 2021.

[7] Karan A, Negandhi H, Nair R, Sharma A, Tiwari R, Zodpey S (2019). Size, composition and distribution of human resource for health in India: new estimates using National Sample Survey and Registry data. BMJ Open.

[8] NITI Aayog. SDG India Index and Dashboard 2019–20. NITI Aayog; 2019.

[9] Ministry of Health and Family Welfare (MoHFW). National Health Policy 2017. Ministry of Health and Family Welfare (MoHFW), Government of India; 2017. https://www.nhp.gov.in/nhpfiles/national_health_policy_2017.pdf. Accessed 19 Aug 2021.

[10] Global Health Workforce Alliance (GHWA), World Health Organization (WHO). A universal truth: no health without a workforce. World Health Organization (WHO); 2014. https://www.who.int/workforcealliance/knowledge/resources/GHWA-a_universal_truth_report.pdf?ua=1. Accessed 19 Aug 2021.

[11] Global Health Workforce Alliance (GHWA). What do we mean by availability, accessibility, acceptability and quality (AAAQ) of the health workforce? https://www.who.int/workforcealliance/media/qa/04/en/. Accessed 19 Aug 2021.

[12] Krishna L, Dharmadhikari S, Zadey S. Scoping review of the state of human resources for health in India . The Duke Student Global Health Review. 2020. https://dsghreview.org/2020/10/08/scoping-review-of-the-state-of-human-resources-for-health-in-india/. Accessed 19 Aug 2021.

[13] Vasa J, Dharmadhikari S, Dubey S, Zadey S. Why are health workers not going to rural areas? : A systematic review of the qualitative and quantitative studies. CUGH; 2020. p. 167. https://www.dropbox.com/s/0pukydme2xbuwmr/6%20CUGH%202020%20eBook_Abstracts_Strengthening%20Health%20Care%20Systems.pdf?dl=0. Accessed 19 Aug 2021.

[14] Vasa J, Dharmadhikari S, Dubey S, Zadey S. Is there a shortage of human resources for health in India and why?: A systematic review. 2020. https://www.crd.york.ac.uk/prospero/display_record.php?RecordID=159464. Accessed 19 Aug 2021.

[15] National Health Portal of India | Gateway to Authentic Health Information. https://www.nhp.gov.in/. Accessed 19 Aug 2021.

[16] Bhore J, Amesur RA, Banerjea AC, Butt AH, Chandrachud RB, Dadabhoy DJR, et al. Report of the Health Survey and Development Committee: Volume 3. Government of India Press; 1946. https://www.nhp.gov.in/sites/default/files/pdf/Bhore_Committee_Report-3.pdf. Accessed 19 Aug 2021.

[17] Reddy KS, Sethi NK, Chatterjee M, Dasgupta J, Garg A, Jain Y, et al. High Level Expert Group Report on Universal Health Coverage for India . New Delhi: Planning Commission of India; 2011. https://nhm.gov.in/images/pdf/publication/Planning_Commission/rep_uhc0812.pdf. Accessed 19 Aug 2021.

[18] Alphabetical Index of N.C.O. 2004 with occupational titles and equivalent code numbers of N.C.O. 1968. https://labour.gov.in/sites/default/files/AlphabeticalIndex.pdf. Accessed 19 Aug 2021.

[19] Ministry of Home Affairs, Government of India. Office of the Registrar General & Census Commissioner, India. https://censusindia.gov.in/. Accessed 19 Aug 2021.

[20] Office of the Registrar General & Census Commissioner, India. Occupational Classification of Main Workers other than Cultivators and Agricultural Labourers by Sex (Table B-18) | Census of India—1981 . 1981. http://lsi.gov.in:8081/jspui/bitstream/123456789/343/1/28232_1981_GET.pdf. Accessed 17 Aug 2021.

[21] Office of the Registrar General & Census Commissioner, India. Occupational Classification of Main Workers other than Cultivators and Agricultural Labourers by Sex (Table B-25) | Census of India—2001. 2001. https://censusindia.gov.in/Tables_Published/B-Series/B-Series_Link/DDW-B25-0000.pdf. Accessed 18 Aug 2021.

[22] Office of the Registrar General & Census Commissioner, India. Occupational classification of main workers other than cultivators and agricultural labourers by sex (Table B-25A) | Census of India—2011 . 2011. https://censusindia.gov.in/2011census/B-series/B_25A.html. Accessed 17 Aug 2021.

[23] Ministry of Statistics and Programme Implementation. Employment and unemployment | national sample survey | National Data Archive. http://microdata.gov.in/nada43/index.php/catalog/EUE. Accessed 19 Aug 2021.

[24] Rao KD, Bhatnagar A, Berman P (2012). So many, yet few: human resources for health in India. Hum Resour Health.

[25] National Sample Survey Office (NSSO). India—employment and unemployment survey, January to December, 1983, 38th Round. 1983. http://microdata.gov.in/nada43/index.php/catalog/49. Accessed 18 Aug 2021.

[26] National Sample Survey Office (NSSO). India—employment and unemployment, July 2004–June 2005, NSS 61st Round. 2005. http://microdata.gov.in/nada43/index.php/catalog/109. Accessed 19 Aug 2021.

[27] National Sample Survey Office (NSSO). India—employment and unemployment, July 2011–June 2012, NSS 68th Round. 2012. http://microdata.gov.in/nada43/index.php/catalog/127. Accessed 17 Aug 2021.

[28] Landrum K, Cotache-Condor CF, Liu Y, Truche P, Robinson J, Thompson N (2021). Global and regional overview of the inclusion of paediatric surgery in the national health plans of 124 countries: an ecological study. BMJ Open.

[29] Citron I, Chokotho L, Lavy C (2016). Prioritisation of surgery in the national health strategic plans of Africa: a systematic review. World J Surg.

[30] Meara JG, Leather AJM, Hagander L, Alkire BC, Alonso N, Ameh EA (2015). Global surgery 2030: evidence and solutions for achieving health, welfare, and economic development. Lancet.

[31] Campbell J, Buchan J, Cometto G, David B, Dussault G, Fogstad H (2013). Human resources for health and universal health coverage: fostering equity and effective coverage. Bull World Health Organ.

[32] Pozo-Martin F, Nove A, Lopes SC, Campbell J, Buchan J, Dussault G (2017). Health workforce metrics pre- and post-2015: a stimulus to public policy and planning. Hum Resour Health.

[33] Craveiro I, Hortale V, de Oliveira APC, Dal Poz M, Portela G, Dussault G (2018). The utilization of research evidence in Health Workforce Policies: the perspectives of Portuguese and Brazilian National Policy-Makers. J Public Health (Oxf).

[34] Statistics Division, Ministry of Health and Family Welfare (MoHFW). Rural Health Statistics (RHS) 2018–2019 . Ministry of Health and Family Welfare (MoHFW), Government of India; 2019. https://main.mohfw.gov.in/sites/default/files/Final%20RHS%202018-19_0.pdf. Accessed 10 Jul 2021.

[35] Hamdan M, Defever M (2003). Human resources for health in Palestine: a policy analysis. Part I: current situation and recent developments. Health Policy.

[36] Rao KD, Shahrawat R, Bhatnagar A (2016). Composition and distribution of the health workforce in India: estimates based on data from the National Sample Survey. WHO South East Asia J Public Health.

[37] Hazarika I (2013). Health workforce in India: assessment of availability, production and distribution. WHO South East Asia J Public Health.

[38] Chandra S. Indian healthcare’s inconvenient truth. The Hindu Business Line. 2018. https://www.thehindubusinessline.com/opinion/indian-healthcares-inconvenient-truth/article22259516.ece. Accessed 19 Aug 2021.

[39] van de Pas R, Veenstra A, Gulati D, Van Damme W, Cometto G (2017). Tracing the policy implementation of commitments made by national governments and other entities at the Third Global Forum on Human Resources for Health. BMJ Glob Health.

[40] World Health Organization (WHO). Increasing access to health workers in remote and rural areas through improved retention: Global Policy Recommendations. World Health Organization (WHO); 2010. https://www.who.int/publications/i/item/increasing-access-to-health-workers-in-remote-and-rural-areas-through-improved-retention. Accessed 19 Aug 2021. 23741785

[41] Zadey S, Dubey S. Helping doctors reach rural India. Global Health NOW. 2021. https://www.globalhealthnow.org/2021-02/helping-doctors-reach-rural-india. Accessed 22 Aug 2021.

[42] NITI Aayog. SDG India: Index & Dashboard 2020–21 . NITI Aayog, Government of India (GoI); 2021. https://www.niti.gov.in/writereaddata/files/SDG_3.0_Final_04.03.2021_Web_Spreads.pdf. Accessed 10 Oct 2021.

[43] Karan A, Negandhi H, Hussain S, Zapata T, Mairembam D, De Graeve H (2021). Size, composition and distribution of health workforce in India: why, and where to invest?. Hum Resour Health.

[44] Mazziotta M, Pareto A (2016). On a generalized non-compensatory composite index for measuring socio-economic phenomena. Soc Indic Res.

[45] de Loë RC, Melnychuk N, Murray D, Plummer R (2016). Advancing the state of policy delphi practice: a systematic review evaluating methodological evolution, innovation, and opportunities. Technol Forecast Soc Change.

[46] World Health Organization. The World Health Report 2006: working together for health (world Health Reports). World Health Organization; 2006.

