# Supplementary material for: Do health policies address the availability, accessibility, acceptability, and quality of human resources for health? Analysis over three decades of National Health Policy of India
Source: Hum Resour Health. 2021 Nov 13;19:139. doi: 10.1186/s12960-021-00681-1 (PMC8590377; doi:10.1186/s12960-021-00681-1)
Supplement: Supplementary file 1 — Additional file 1. 1.a. References for HRH strategies and actions in the AAAQ framework. Legend—HRH: Human Resources for Health, AAAQ: Availability, Accessibility, Acceptability, Quality. 1.b. Methods Supplement. Description of data: The file contains equations for calculations of R-1 (Bhore thresholds) and R-2 (HLEG thresholds) at health center levels and P for pre-NHPI census—1981, 2001, and 2011 years along with considerations and limitations used for calculations. [file 12960_2021_681_MOESM1_ESM.docx]

**ADDITIONAL FILE 1 - Methods Supplement**

**(a) References for HRH strategies and actions used in AAAQ framework**

| **Dimension** | **Strategy** | **Action** | **References** |
| --- | --- | --- | --- |
| Availability | Increase HRH production | Establish new training institutes | [(1)](https://sciwheel.com/work/citation?ids=11560576&pre=&suf=&sa=0) |
|  |  | Increase number of seats in existing training institutes | [(1)](https://sciwheel.com/work/citation?ids=11560576&pre=&suf=&sa=0) |
|  |  | Create new HRH cadres | [(2)](https://sciwheel.com/work/citation?ids=10166127&pre=&suf=&sa=0) |
|  | Task shifting and task sharing | - | [(3)](https://sciwheel.com/work/citation?ids=8158506&pre=&suf=&sa=0) |
|  | Recruit HRH from foreign countries | - | [(4)](https://sciwheel.com/work/citation?ids=11575686&pre=&suf=&sa=0) |
|  | Retain HRH within the country | Reduce immigration | [(5)](https://sciwheel.com/work/citation?ids=7627464&pre=&suf=&sa=0) |
|  |  | Reduce attrition | [(6)](https://sciwheel.com/work/citation?ids=10166124&pre=&suf=&sa=0) |
|  | Develop information systems and tools to measure and monitor the availability of HRH | - | [(1)](https://sciwheel.com/work/citation?ids=11560576&pre=&suf=&sa=0) |
| Accessibility | Increase HRH production in the underserved areas | Establish new training institutes in underserved areas | [(1,7)](https://sciwheel.com/work/citation?ids=11575699,11560576&pre=&pre=&suf=&suf=&sa=0,0) |
|  |  | Create a new HRH cadre specifically for underserved areas | [(1,2)](https://sciwheel.com/work/citation?ids=10166127,11560576&pre=&pre=&suf=&suf=&sa=0,0) |
|  | Task shifting and multitasking of HRH cadre in underserved areas | - | [(8)](https://sciwheel.com/work/citation?ids=11575703&pre=&suf=&sa=0) |
|  | Attract and retain HRH from surplus sector/area/level of care/system of medicine to underserved areas | Mandatory rural postings | [(6,9)](https://sciwheel.com/work/citation?ids=10166124,10164351&pre=&pre=&suf=&suf=&sa=0,0) |
|  |  | Remove administrative barrier in recruitment | [(10)](https://sciwheel.com/work/citation?ids=4989408&pre=&suf=&sa=0) |
|  |  | Identify groups/individuals motivated to work in underserved areas | [(11)](https://sciwheel.com/work/citation?ids=11575720&pre=&suf=&sa=0) |
|  |  | Provide financial/non-financial incentives | [(10,12,13)](https://sciwheel.com/work/citation?ids=4989408,11575738,11575740&pre=&pre=&pre=&suf=&suf=&suf=&sa=0,0,0) |
|  |  | Remove professional isolation | [(10)](https://sciwheel.com/work/citation?ids=4989408&pre=&suf=&sa=0) |
|  |  | Mainstream and integrate indigenous HRH in underserved areas | [(8)](https://sciwheel.com/work/citation?ids=11575703&pre=&suf=&sa=0) |
|  | Tele-consultation | - | [(14)](https://sciwheel.com/work/citation?ids=8874459&pre=&suf=&sa=0) |
|  | Develop information systems and tools to measure and monitor the geographical distribution of HRH | - | [(1)](https://sciwheel.com/work/citation?ids=11560576&pre=&suf=&sa=0) |
| Acceptability | Create HRH closer to the community | Give preference to local candidates in training institutes | [(15)](https://sciwheel.com/work/citation?ids=10166162&pre=&suf=&sa=0) |
|  | Develop sociocultural aspects in HRH | Emphasize sociocultural aspects in the medical curriculum | [(16)](https://sciwheel.com/work/citation?ids=8686460&pre=&suf=&sa=0) |
|  |  | Induction training | [(17)](https://sciwheel.com/work/citation?ids=10164361&pre=&suf=&sa=0) |
|  | Deploy HRH in their local community | - | [(10)](https://sciwheel.com/work/citation?ids=4989408&pre=&suf=&sa=0) |
|  | Compose and recruit HRH representative of gender/age/religion of population being served | - | [(18)](https://sciwheel.com/work/citation?ids=11560520&pre=&suf=&sa=0) |
|  | Expand cadre with high local acceptance preferentially | - | [(19)](https://sciwheel.com/work/citation?ids=11575780&pre=&suf=&sa=0) |
|  | Create an appropriate skill mix of cadres | - | [(18)](https://sciwheel.com/work/citation?ids=11560520&pre=&suf=&sa=0) |
| Quality | Produce quality HRH | Changing curriculum to suit all levels of care | [(20)](https://sciwheel.com/work/citation?ids=11576060&pre=&suf=&sa=0) |
|  |  | Improve training of HRH cadres | [(12)](https://sciwheel.com/work/citation?ids=11575738&pre=&suf=&sa=0) |
|  |  | Develop interpersonal/soft skills in HRH cadres | [(12)](https://sciwheel.com/work/citation?ids=11575738&pre=&suf=&sa=0) |
|  |  | Professional councils for HRH | [(21)](https://sciwheel.com/work/citation?ids=11576295&pre=&suf=&sa=0) |
|  |  | Standard licensing exam for all cadre | [(22)](https://sciwheel.com/work/citation?ids=11576354&pre=&suf=&sa=0) |
|  | Formal training courses for unqualified HRH | - | [(23)](https://sciwheel.com/work/citation?ids=5715273&pre=&suf=&sa=0) |
|  | Grievance redressal and feedback system for patients | - | [(24)](https://sciwheel.com/work/citation?ids=2645778&pre=&suf=&sa=0) |
|  | Maintain quality of HRH | CME and training to HRH | [(25)](https://sciwheel.com/work/citation?ids=9516129&pre=&suf=&sa=0) |
|  |  | Establish policy/rules for promotion, transfer, salary, etc. for all HRH | [(1,10)](https://sciwheel.com/work/citation?ids=4989408,11560576&pre=&pre=&suf=&suf=&sa=0,0) |
|  |  | Timely assessment and performance-based incentives | [(26)](https://sciwheel.com/work/citation?ids=1136776&pre=&suf=&sa=0) |
|  | Conduct meetings to review common medical errors | - | [(27)](https://sciwheel.com/work/citation?ids=11845937&pre=&suf=&sa=0) |

**(b) Calculation of ‘P’ and ‘R’:**

**Definitions:**

P_cadre_year_ =Number of HRH cadre personnel present per 100,000 population in the given year

= Present HRH cadre density per 100,000 in the given year

R_cadre_threshold_ = Number of HRH cadre personnel required for 100,000 population using a

prescribed threshold

= Required HRH cadre density per 100,000 as per prescribed threshold

Note: P_cadre_year_ and R_cadre_threshold_ are equivalent to ‘P’ and ‘R’ in the manuscript.

P_cadre_year_ was calculated for six cadres allopathic doctors (referred to as doctors in rest of the paper), Ayurveda, Yoga and Naturopathy, Unani, Siddha and Homeopathy (AYUSH) practitioners, nurses, auxiliary nurse-midwives (ANMs), dentists, and pharmacists at national levels for census years immediately preceding the release of NHPI - 1981, 2001, and 2011 (i.e. pre-NHPI census years). R_cadre_threshold_ was computed for the same cadres using thresholds prescribed by the Bhore (Health Survey and Development) in 1946 (R-1) [(28)](https://sciwheel.com/work/citation?ids=11560612&pre=&suf=&sa=0) and High-Level Expert Group (HLEG) (R-2) [(1)](https://sciwheel.com/work/citation?ids=11560576&pre=&suf=&sa=0) Committees in 2011.

**Calculation of R_cadre_threshold_ :**

The Bhore Committee Volume III [(28)](https://sciwheel.com/work/citation?ids=11560612&pre=&suf=&sa=0) mentions requirement thresholds for cadres at three health-center levels - primary health unit (PHU), secondary health unit (SHU), and district headquarter (DHQ). The HLEG Committee [(3)](https://paperpile.com/c/HGBkK7/NNfz) mentions requirement thresholds for cadres at six health-center levels - subcenter (SC), primary health center (PHC), community health center (CHC), sub-district hospital (SDH), district hospital (DH), and medical college teaching hospital (MCH). In both committees, each health center caters to a particular population and each health center has a cadre-wise threshold of personnel required.

Using these two, the cadre-wise threshold (T_cadre_report_centre_) of personnel required for a particular population can be derived and can be standardized for 100,000 population (R_cadre_report_centre_).

In the Bhore Committee report,

1. A PHU caters to 10000 population, therefore, $R_{cadre\_Bhore\_PHU}= \frac{100000 \times T_{cadre\_Bhore\_PHU}}{10000}$
2. A SHU caters to 300000 population, therefore, $R_{cadre\_Bhore\_SHU}= \frac{100000 \times T_{cadre\_Bhore\_SHU}}{300000}$
3. A DHQ caters to a district, therefore, $R_{cadre\_Bhore\_DHQ}= \frac{100000 \times T_{cadre\_Bhore\_DHQ}}{average district population}$

* Note - $average district population \left( ADP \right)= \frac{{ADP}_{1981}+{ADP}_{2001}+{ADP}_{2011}}{3}$

$$R_{cadre\_Bhore}= R_{cadre\_Bhore\_PHU}+ R_{cadre\_Bhore\_SHU}+ R_{cadre\_Bhore\_DHQ}$$

In the HLEG report,

1. An SC caters to 3000 population, therefore $R_{cadre\_HLEG\_SC}= \frac{100000 \times T_{cadre\_HLEG\_SC}}{3000}$
2. A PHC caters to 20000 population, therefore $R_{cadre\_HLEG\_PHC}= \frac{100000 \times T_{cadre\_HLEG\_PHC}}{20000}$
3. A CHC caters to 80000 population, therefore $R_{cadre\_HLEG\_CHC}= \frac{100000 \times T_{cadre\_HLEG\_CHC}}{80000}$
4. An SDH caters to 1000000 population, therefore $R_{cadre\_HLEG\_SDH}= \frac{100000 \times T_{cadre\_HLEG\_SDH}}{1000000}$
5. A DH caters to 1000000 population, therefore $R_{cadre\_HLEG\_DH}= \frac{100000 \times T_{cadre\_HLEG\_DH}}{1000000}$
6. An MCH caters to 2500000 population, therefore $R_{cadre\_HLEG\_MCH}= \frac{100000 \times T_{cadre\_HLEG\_MCH}}{2500000}$

$R_{cadre\_HLEG}= R_{cadre\_HLEG\_SHC}+ R_{cadre\_HLEG\_PHC}+ R_{cadre\_HLEG\_CHC}+ R_{cadre\_HLEG\_SDH}+ R_{cadre\_HLEG\_DH}+ R_{cadre\_HLEG\_MCH}$

Considerations and limitations:

1. In R_pharmacist_Bhore_, pharmacists include pharmacists and compounders [(28)](https://sciwheel.com/work/citation?ids=11560612&pre=&suf=&sa=0). Compounders were a pharmaceutical cadre during the peri-Independence era, with 1 or 2 years of qualification in Pharmacy. Compounders are included with pharmacists here as all compounder posts were to be replaced by pharmacists eventually when enough pharmacists become available.
2. In R_pharmacist_HLEG,_ pharmacists include allopathic and AYUSH pharmacists.
3. In R_nurse_Bhore_ and R_nurse_HLEG_ nurses include staff nurses, head nurses, matrons, public health nurses, and district public health nurses.
4. In R_doctor_Bhore_ and R_doctor_HLEG_ doctors include both graduates and postgraduates in allopathic medicine.
5. The Bhore committee did not mention the number of AYUSH at any center level. Therefore, R_AYUSH_Bhore_ cannot be calculated.

**Calculation of P_cadre_year_ :**

We extracted the absolute number of male and female personnel of each cadre for rural, urban, and total groups at the national level from the Census of India [(29)](https://sciwheel.com/work/citation?ids=11560729&pre=&suf=&sa=0) for years -1981, 2001, and 2011. National Sample Survey (NSS) rounds on ‘Employment and Unemployment’ [(30)](https://sciwheel.com/work/citation?ids=11560719&pre=&suf=&sa=0) were used to extract the qualified HRH data. We compared the educational achievements of self-reported health workers in the 38^th^ (January to December 1983) [(31)](https://sciwheel.com/work/citation?ids=11560744&pre=&suf=&sa=0), 61^st^ (July 2004-June 2005) [(32)](https://sciwheel.com/work/citation?ids=11560948&pre=&suf=&sa=0), and 68^th^ (July 2011 to June 2012) [(33)](https://sciwheel.com/work/citation?ids=11560730&pre=&suf=&sa=0) NSS rounds with the required qualifications as recommended by registration institutes and councils for each cadre e.g. Medical Council of India for allopathic doctors and Indian Nursing Council for nurses, etc [(34)](https://sciwheel.com/work/citation?ids=10072486&pre=&suf=&sa=0). The absolute numbers were used to get cadre-wise present HRH densities (P_cadre_year_) per 100,000 for 1981, 2001, 2011 using the respective census populations.

For national,

$P_{cadre\_national\_year}= \frac{absolute number of personnel under the cadre at national level}{total population in the year} \times100000$

For rural,

$P_{cadre\_rural\_year}= \frac{absolute number of personnel under the cadre at rural level}{total rural population in the year} \times100000$

For urban,

$P_{cadre\_urban\_year}= \frac{absolute number of personnel under the cadre at urban level}{total urban population in the year} \times100000$

**Deficit Indices:**

Using P_cadre_year_ and R_cadre_threshold_ we calculated deficit indices for four dimensions, Availability deficit (AvD), Accessibility deficit (AsD), Acceptability deficit (ApD), and quality deficit (QD) as given in the manuscript.

**References:**

[1. Reddy KS, Sethi NK, Chatterjee M, Dasgupta J, Garg A, Jain Y, et al. High Level Expert Group Report on Universal Health Coverage for India [Internet]. New Delhi: Planning Commission of India; 2011 [cited 2021 Aug 19]. Available from: https://nhm.gov.in/images/pdf/publication/Planning_Commission/rep_uhc0812.pdf](https://sciwheel.com/work/bibliography/11560576)

[2. Lisam S, Nandi S, Kanungo K, Verma P, Mishra JP, Mairembam DS. Strategies for attraction and retention of health workers in remote and difficult-to-access areas of Chhattisgarh, India: Do they work? Indian J Public Health. 2015 Sep;59(3):189–95.](https://sciwheel.com/work/bibliography/10166127)

[3. Dawson AJ, Buchan J, Duffield C, Homer CSE, Wijewardena K. Task shifting and sharing in maternal and reproductive health in low-income countries: a narrative synthesis of current evidence. Health Policy Plan. 2014 May;29(3):396–408.](https://sciwheel.com/work/bibliography/8158506)

[4. Dash S, Bhan A. Indian healthcare needs foreign medical graduates. Draft rules do just the opposite. The Print [Internet]. 2021 Jun 29 [cited 2021 Aug 22]; Available from: https://theprint.in/opinion/indian-healthcare-needs-foreign-medical-graduates-draft-rules-do-just-the-opposite/686226/](https://sciwheel.com/work/bibliography/11575686)

[5. Walton-Roberts M, Runnels V, Rajan SI, Sood A, Nair S, Thomas P, et al. Causes, consequences, and policy responses to the migration of health workers: key findings from India. Hum Resour Health. 2017 Apr 5;15(1):28.](https://sciwheel.com/work/bibliography/7627464)

[6. Goel S, Angeli F, Bhatnagar N, Singla N, Grover M, Maarse H. Retaining health workforce in rural and underserved areas of India: What works and what doesn’t? A critical interpretative synthesis. Natl Med J India. 2016 Aug;29(4):212–8.](https://sciwheel.com/work/bibliography/10166124)

[7. Brahmapurkar KP, Zodpey SP, Sabde YD, Brahmapurkar VK. The need to focus on medical education in rural districts of India. Natl Med J India. 2018 Jun;31(3):164–8.](https://sciwheel.com/work/bibliography/11575699)

[8. Balsari S, Phadke M, Simon G, Goyal R, Mulholland I. Task Shifting in Healthcare: Reframing the AYUSH Debate [Internet]. Harvard University South Asia Institute; 2017 [cited 2021 Aug 23]. Available from: https://cdn2.sph.harvard.edu/wp-content/uploads/sites/114/2017/12/Task-Shifting-in-Indian-Healthcare-Jan-2017.pdf](https://sciwheel.com/work/bibliography/11575703)

[9. Dharmadhikari S, Dubey S, Zadey S. Why Bonded Service for Doctors Has Seen Only Limited Success - The Wire Science [Internet]. Science. The Wire. 2020 [cited 2020 Dec 12]. Available from: https://science.thewire.in/health/why-bonded-service-for-doctors-has-seen-only-limited-success/](https://sciwheel.com/work/bibliography/10164351)

[10. Rajbangshi PR, Nambiar D, Choudhury N, Rao KD. Rural recruitment and retention of health workers across cadres and types of contract in north-east India: A qualitative study. WHO South East Asia J Public Health. 2017;6(2):51–9.](https://sciwheel.com/work/bibliography/4989408)

[11. Zadey S, Dubey S. Helping Doctors Reach Rural India  [Internet]. Global Health NOW. 2021 [cited 2021 Aug 22]. Available from: https://www.globalhealthnow.org/2021-02/helping-doctors-reach-rural-india](https://sciwheel.com/work/bibliography/11575720)

[12. Nair KS. Health workforce in India: opportunities and challenges. Int J Community Med Public Health. 2019 Sep 26;6(10):4596.](https://sciwheel.com/work/bibliography/11575738)

[13. George G, Rhodes B. Is there a financial incentive to immigrate? Examining of the health worker salary gap between India and popular destination countries. Hum Resour Health. 2017 Oct 19;15(1):74.](https://sciwheel.com/work/bibliography/11575740)

[14. Zanaboni P, Scalvini S, Bernocchi P, Borghi G, Tridico C, Masella C. Teleconsultation service to improve healthcare in rural areas: acceptance, organizational impact and appropriateness. BMC Health Serv Res. 2009 Dec 18;9:238.](https://sciwheel.com/work/bibliography/8874459)

[15. Sundararaman T, Gupta G. Indian approaches to retaining skilled health workers in rural areas. Bull World Health Organ. 2011 Jan 1;89(1):73–7.](https://sciwheel.com/work/bibliography/10166162)

[16. Ghosh SK, Biswas S. Diversity in medical education: the Indian Paradox. Med Educ Online. 2014 Dec 10;19:26395.](https://sciwheel.com/work/bibliography/8686460)

[17. Sharma DC. India still struggles with rural doctor shortages. Lancet. 2015 Dec 12;386(10011):2381–2.](https://sciwheel.com/work/bibliography/10164361)

[18. Global Health Workforce Alliance (GHWA), World Health Organization (WHO). A Universal Truth: No Health Without a Workforce [Internet]. World Health Organization (WHO); 2014 [cited 2021 Aug 19]. Available from: https://www.who.int/workforcealliance/knowledge/resources/GHWA-a_universal_truth_report.pdf?ua=1](https://sciwheel.com/work/bibliography/11560520)

[19. Grant M, Wilford A, Haskins L, Phakathi S, Mntambo N, Horwood CM. Trust of community health workers influences the acceptance of community-based maternal and child health services. Afr J Prim Health Care Fam Med. 2017 May 29;9(1):e1–8.](https://sciwheel.com/work/bibliography/11575780)

[20. Task Force on Medical Education. Report of the Task Force on Medical Education for the National Rural Health Mission [Internet]. Ministry of Health and Family Welfare (MoHFW), Government of India (GoI); 2007 [cited 2021 Aug 23]. Available from: https://ideas.repec.org/p/ess/wpaper/id1082.html](https://sciwheel.com/work/bibliography/11576060)

[21. Lal DK. Regulation of health workforce in India. Rev Direito Sanit. 2018 Dec 11;19(2):87–130.](https://sciwheel.com/work/bibliography/11576295)

[22. Sutherland K, Leatherman S. Does certification improve medical standards? BMJ. 2006 Aug 26;333(7565):439–41.](https://sciwheel.com/work/bibliography/11576354)

[23. Mohanan M, Hay K, Mor N. Quality of health care in india: challenges, priorities, and the road ahead. Health Aff (Millwood). 2016 Oct 1;35(10):1753–8.](https://sciwheel.com/work/bibliography/5715273)

[24. Reader TW, Gillespie A, Roberts J. Patient complaints in healthcare systems: a systematic review and coding taxonomy. BMJ Qual Saf. 2014 Aug;23(8):678–89.](https://sciwheel.com/work/bibliography/2645778)

[25. Das S, Shah M, Mane A, Goyal V, Singh V, Lele J. Accreditation in india: pathways and mechanisms. Journal of European CME. 2018 Apr 4;7(1):1454251.](https://sciwheel.com/work/bibliography/9516129)

[26. Rosenthal MB, Fernandopulle R, Song HR, Landon B. Paying for quality: providers’ incentives for quality improvement. Health Aff (Millwood). 2004 Apr;23(2):127–41.](https://sciwheel.com/work/bibliography/1136776)

[27. Evidence and Information for Policy Department of Health Service Provision, World Health Organization (WHO). Quality and accreditation in health care services: A global review [Internet]. Geneva, Switzerland: World Health Organization (WHO); 2003 [cited 2021 Oct 12]. Available from: https://www.who.int/hrh/documents/en/quality_accreditation.pdf](https://sciwheel.com/work/bibliography/11845937)

[28. Bhore J, Amesur RA, Banerjea AC, Butt AH, Chandrachud RB, Dadabhoy DJR, et al. Report of the Health Survey and Development Committee: Volume 3 [Internet]. Government of India Press; 1946 [cited 2021 Aug 19]. Available from: https://www.nhp.gov.in/sites/default/files/pdf/Bhore_Committee_Report-3.pdf](https://sciwheel.com/work/bibliography/11560612)

[29. Ministry of Home Affairs, Government of India. Office of the Registrar General & Census Commissioner, India [Internet]. [cited 2021 Aug 19]. Available from: https://censusindia.gov.in/](https://sciwheel.com/work/bibliography/11560729)

[30. Ministry of Statistics and Programme Implementation. Employment and Unemployment | National Sample Survey | National Data Archive  [Internet]. [cited 2021 Aug 19]. Available from: http://microdata.gov.in/nada43/index.php/catalog/EUE](https://sciwheel.com/work/bibliography/11560719)

[31. National Sample Survey Office (NSSO). India - Employment and Unemployment Survey, January to December, 1983, 38th Round [Internet]. 1983 [cited 2021 Aug 18]. Available from: http://microdata.gov.in/nada43/index.php/catalog/49](https://sciwheel.com/work/bibliography/11560744)

[32. National Sample Survey Office (NSSO). India - Employment and Unemployment, July 2004 - June 2005, NSS 61st Round [Internet]. 2005 [cited 2021 Aug 19]. Available from: http://microdata.gov.in/nada43/index.php/catalog/109](https://sciwheel.com/work/bibliography/11560948)

[33. National Sample Survey Office (NSSO). India - Employment and Unemployment, July 2011- June 2012, NSS 68th Round [Internet]. 2012 [cited 2021 Aug 17]. Available from: http://microdata.gov.in/nada43/index.php/catalog/127](https://sciwheel.com/work/bibliography/11560730)

[34. Karan A, Negandhi H, Nair R, Sharma A, Tiwari R, Zodpey S. Size, composition and distribution of human resource for health in India: new estimates using National Sample Survey and Registry data. BMJ Open. 2019 May 27;9(4):e025979.](https://sciwheel.com/work/bibliography/10072486)
